# Ensemble Niche Modelling Projects Net Suitability Gain and Eastward Range Expansion for the Namaqua Dove (*Oena capensis*) in Anatolia Under Climate Change

**DOI:** 10.3390/ani16142238

**Published:** 2026-07-19

**Authors:** Bekir Kabasakal

**Affiliations:** Vocational School of Health Services, Antalya Bilim University, 07190 Antalya, Türkiye; kabasakalbekir@gmail.com

**Keywords:** *Oena capensis*, Namaqua Dove, ecological niche model, ensemble forecasting, niche dynamics, range expansion, non-analogue climate

## Abstract

Climate-driven range shifts are redistributing biodiversity, yet the niche dynamics of Afrotropical and Saharo-Arabian species expanding into the Palearctic remain poorly quantified. The Namaqua Dove (*Oena capensis*), an arid-zone columbid at the Palearctic margin, was used to assess current and future climatic suitability in Türkiye and to compare its colonised and native niches. An ensemble niche model was fitted from occurrence and climate data, evaluated by cross-validation, and projected to 2070 and 2100 under three emission scenarios and five climate models. Suitability was predicted chiefly by precipitation, with low winter temperature as a secondary constraint, and roughly 92,000 km^2^ is already climatically suitable. In environmental space, the colonised records formed a contained, cool-margin subset of the native niche, indicating partial filling rather than expansion into novel climate. Low niche overlap, non-significant similarity tests, and a failed native-model transfer indicate a non-equilibrium, still-establishing front rather than full niche conservatism. The suitable area increased under every scenario and shifted predominantly eastward, with a north-eastward component, rather than poleward. The eastward pattern reflects the geometry of the suitable climate across Anatolia rather than an intrinsic directional preference. Because most of the projected climate has no present-day analogue, the direction of change is better supported than its magnitude, though both are conditioned by this extrapolation. The projected magnitude is reported as scenario illustration rather than forecast. These results indicate that suitability for *O. capensis* in Türkiye is shaped jointly by precipitation and winter temperature, with colonisation tracking suitable climate rather than shifting into novel conditions.

## Simple Summary

The Namaqua Dove (*Oena capensis*), a small ground-dwelling dove of Africa and Arabia, is increasingly recorded further north and has recently begun to breed in Türkiye. Occurrence records and climate data were combined in an ensemble of statistical models to map where climate is currently suitable for the species, how this may change as the climate warms, and how the climate it occupies in Türkiye compares with that of its native range. Southern and central Türkiye are already suitable, and the suitable area is projected to expand under warming, predominantly eastward and north-eastward rather than due north. So far, the birds occupy only the cooler, drier edge of the climatic conditions used in their native range, consistent with an early, still-spreading front that is using the suitable climate rather than adapting to genuinely new conditions. Because much of the future climate projected for Türkiye has few present-day parallels, the models indicate the likely direction of spread more reliably than its eventual extent. These findings point to where the species may appear next and help target its monitoring.

## 1. Introduction

As the climate warms, many species are tracking shifting conditions poleward in latitude and upward in elevation, redistributing biodiversity worldwide [1,2,3]. The reported rates of these shifts vary widely among taxa and regions, and they often depart from the simple poleward expectation [4,5]. Understanding where suitable climate exists now, how it will move, and whether a species retains or modifies its climatic requirements during expansion is central to anticipating future distributions [6,7]. 

Birds are among the most responsive taxa to recent climate change, and avian range margins have shifted measurably across Europe and beyond [8,9]. Within the eastern Mediterranean, warm-affiliated birds have similarly extended their ranges in recent decades. The White-spectacled Bulbul (*Pycnonotus xanthopygos*), for example, spread along the Mediterranean coast of Türkiye and enlarged its breeding range during the twentieth century [10,11], and niche models project it to expand further northward under warming [12]. Range expansions at the leading edge are of particular interest because they reveal the climatic and biotic conditions under which a species can colonise new ground [13]. When a species expands from a warm-adapted source region into a cooler one, two contrasting expectations arise. Under niche conservatism, the colonising population occupies climatic conditions equivalent to those of its native range, and expansion reflects the geographic movement of the suitable climate [14,15]. Under niche shift, the population establishes in novel climatic space, and expansion reflects ecological or evolutionary change. Distinguishing these alternatives requires explicit, quantitative comparison of native and colonised niches rather than qualitative inference [16,17].

The Namaqua Dove (*Oena capensis*, Columbidae) offers an informative case. The species is a small, primarily granivorous, ground-foraging columbid native to sub-Saharan Africa, the Arabian Peninsula, and Madagascar, where it occupies arid and semi-arid open habitats [18]. The species is also an efficient evaporative cooler, tolerating air temperatures well above 50 °C [19], so that at cool range margins, low winter temperature, rather than summer heat, is the more likely climatic limit. Over recent decades, it has been recorded progressively further north along the Palearctic margin. The first national record for Türkiye dates to 2005 [20], and further northward records have been reported subsequently [21,22]. A country-wide analysis of community-science records subsequently identified a significant increase in the detection of the species within Türkiye between 2001 and 2022 (records accruing only after the first national record in 2005) [23]. Over this period, the species is reported to have become established and to have bred in Anatolia [23], whereas breeding is long-established along the adjacent Palearctic margin in Israel, Arabia, Iran and Iraq [22]. Such records place an Afrotropical and Saharo-Arabian species at the threshold of the Western Palearctic [22], a region where biogeographic refugia and dispersal corridors have shaped resident bird lineages such as the partridge *Alectoris* [24]. Because Anatolia lies at this Palearctic threshold, where an arid-zone colonist meets a cold-winter interior, it offers an informative test of whether the colonised niche remains within the native envelope. This raises the question of how much of Anatolia is climatically suitable and whether suitability will expand under continued warming. The ecological significance of such expansions extends beyond biogeography, because non-native and range-expanding birds can affect recipient ecosystems, including through competition and disease transmission [25]. Because the species is a native range-expander tracking the suitable climate rather than a classic non-native invader, delimiting where such a colonist could establish is also of applied relevance. Despite growing observational evidence of northward movement, the climatic determinants of suitability for *O. capensis* in Türkiye have not yet been quantified with a formal niche model, and the niche dynamics of its expansion have not been tested.

Ecological niche models, also termed species distribution models, relate occurrence records to environmental predictors to estimate suitability across space and time [6,7]. Single-algorithm predictions can vary substantially, so ensemble approaches that combine multiple algorithms are now widely recommended to characterise and reduce predictive uncertainty [26,27,28]. Reliable application further depends on careful occurrence cleaning, spatial thinning to reduce sampling bias, predictor selection that limits collinearity, spatially structured cross-validation, threshold-independent evaluation, and explicit reporting of extrapolation into non-analogue climate [29,30,31,32]. Each of these elements bears directly on whether projected range changes can be trusted.

Within Türkiye, niche modelling has been applied to several taxa under climate change, including the brown bear, breeding vultures, freshwater turtles, and disease vectors [33,34,35,36], and, for a migratory bird, the Common Cuckoo (*Cuculus canorus*) [37]. These studies establish the regional relevance of the approach and a basis for comparison. They also illustrate that responses differ in direction and magnitude across species, which makes taxon-specific assessment necessary. For an expanding Afrotropical columbid at the Palearctic margin, no comparable quantitative assessment exists yet for Türkiye.

Two gaps, therefore, motivate this study. The first is empirical. The climatic suitability of Türkiye for *O. capensis*, its projected change under future scenarios, and the geometry of its range shift remain unquantified. The second is conceptual and methodological. Whether the northward expansion of this species reflects niche conservatism or niche shift has not been tested with a formal niche-overlap framework, and the degree to which projections rely on the non-analogue climate has not been reported.

In this study, therefore, the climatic niche of *O. capensis* was modelled to address the following four questions: (i) which climatic variables best predict current suitability in Türkiye and how much of the country is climatically suitable; (ii) how the suitable area will change by 2070 and 2100 under three emission scenarios and five global climate models; (iii) whether the colonised niche is conserved relative to the native niche or has shifted; and (iv) in which direction the suitable climate is projected to shift, and whether that shift departs from a purely poleward expectation. In line with these questions, four a priori hypotheses were formulated. First, precipitation and low winter temperature limit suitability, given the arid-zone affinity of the species and the cold winters of interior Anatolia. Second, the suitable area increases on balance under warming. Third, the colonised niche remains largely within the native niche rather than shifting into novel climate, by analogy with evidence that many range-expanding and introduced animals conserve much of their native climatic niche [15,38]. Fourth, the projected shift is predominantly eastward rather than purely poleward, given the strong west–east and coastal–interior climatic gradients of Anatolia.

## 2. Materials and Methods

### 2.1. Occurrence Data and Study Design

A correlative ensemble niche model was built for *Oena capensis* and projected to current and future climates of Türkiye. The full workflow was scripted in R and is reported following the ODMAP protocol for reproducible niche-model documentation ([32], Table S1). Analyses were performed in R (version 4.5.3) with the packages biomod2 v.4.3-4-5, ENMeval v.2.0.5.2, ecospat v.4.1.3, spThin v.0.2.0, CoordinateCleaner v.3.0.1, spdep v.1.4.2, and maxnet v.0.1.4. Spatial operations and figures used sf v.1.1-0, terra v.1.9-11, raster v.3.6-32, ggplot2 v.4.0.3, tidyterra v.1.1.0, ggspatial v.1.1.10, rnaturalearth v.1.2.0, and patchwork v.1.3.2. A fixed random seed (42) was set so that stochastic steps, including pseudo-absence sampling and replicate data splits, are reproducible. Geographic operations used a Türkiye-centred Lambert azimuthal equal-area projection (latitude of origin 39° N, central meridian 35° E) so that areal statistics are computed on an equal-area grid. The land area of Türkiye used for proportional statistics was 783,562 km^2^.

Occurrence records were assembled from the Global Biodiversity Information Facility and supplemented with georeferenced records from the regional literature [39]. Records were retrieved in temporal chunks to capture the full available series, then combined into a single dataset spanning the calibration window 1950–2026. Because the climate predictors represent the 1981–2010 reference period, records from earlier decades are associated with a later climate normal. This temporal mismatch is common in broad-extent niche models and is expected to add noise to, rather than systematically bias, the fitted climate–occurrence relationships.

Raw records were cleaned with a sequence of automated and expert checks. Seven tests from CoordinateCleaner were applied, including flags for records at country and province centroids, capitals, biodiversity-institution coordinates, equal latitude and longitude, zero coordinates, and points falling in the sea [40]. Geographic outliers were also removed using a distance-based criterion. Duplicate records were removed, and records were pre-thinned to one per environmental grid cell to limit duplication at the modelling resolution. To reduce spatial sampling bias, distance-based thinning was applied that retained a single record within a 5 km radius, using 100 thinning repetitions and keeping the repetition that retained the most records [41].

**Figure 1:**
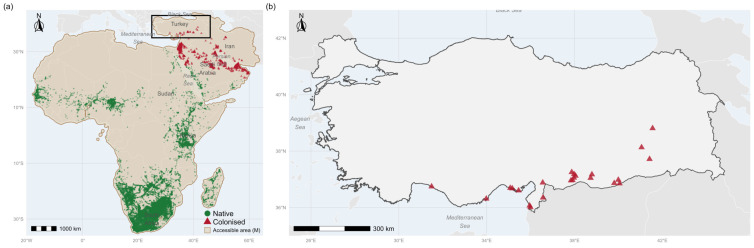
Study area. Native (green) and colonised (vermilion) occurrence records of *Oena capensis* over the calibration area (accessible area M, from which model background was drawn). (**a**) Global (Lambert azimuthal equal-area projection) and (**b**) Türkiye. Base map from Natural Earth (rnaturalearth).

Records were partitioned into native and colonised sets to characterise both ranges and to test niche dynamics (Figure 1). Assignment used a multi-tier geographic scheme refined by expert correction. Records from southwestern Arabia and the Dhofar region were treated as part of the native range, consistent with the recognised native distribution of the species. Records representing isolated vagrants beyond the contiguous expansion front were removed from the colonised set so that the colonised niche reflects established occurrence rather than transient individuals. Specifically, three tiers were applied. Records within the species’ contiguous African and south-western Arabian native range were assigned to the native set. Records along the northward expansion axis, from the Levant into Anatolia, were assigned to the colonised set, and records isolated from this contiguous front were treated as vagrants and excluded. The full operational classification rule, including the bounding-box and coordinate overrides and the resulting record counts, is given in the Supplementary Methods (Section S1, Table S2).

### 2.2. Environmental Predictors and Climate Scenarios

Baseline climate was represented by the 19 standard bioclimatic variables from CHELSA version 2.1 for the reference period 1981–2010 [42]. Variables were aggregated to a working resolution of 2.5 arc-minutes (approximately 5 km at the equator). Before statistical selection, six bioclimatic variables were excluded a priori (BIO3, BIO8, BIO9, BIO11, BIO18, and BIO19) because these combine temperature and precipitation in ways that can introduce spatial artefacts and hinder transfer to new times and places [43]. Predictor rasters were deduplicated by grid cell prior to selection.

From the remaining candidate variables, multicollinearity was removed using the variance inflation factor procedure in the usdm package, iteratively dropping the variable with the highest factor until all values fell below the conventional threshold of 10 ([44,45], Figure S1). The minimum temperature of the coldest month (BIO6) was forced into the model on a priori physiological grounds, because cold tolerance is expected to set the northern limit of an arid-zone columbid. This decision is stated explicitly so that it can be evaluated by the reader. Retaining a physiologically proximal predictor a priori, rather than relying on statistical selection alone, is an established practice in distribution modelling. Such variables tend to transfer more reliably across space and time, and selection on fit alone can favour ecologically arbitrary predictor sets [46,47,48,49]. Predictor importance is later assessed in two complementary ways—range-wide within the ensemble and specifically within Türkiye with a geographical detector—and these need not identify the same leading variable. This distinction is developed in Section 4.1. The final predictor set comprised the mean diurnal temperature range (BIO2), minimum temperature of the coldest month (BIO6), precipitation of the wettest month (BIO13), precipitation of the driest month (BIO14), and precipitation seasonality (BIO15).

### 2.3. Ensemble Species Distribution Modelling

Because the data are presence-only, a target group background was used to mitigate spatial sampling bias when tuning the MaxEnt model. This background was drawn from records of other Columbidae so that it reflects the same observation process as the focal records [50]. For the biomod2 ensemble, pseudo-absences were generated by random selection, with 10,000 drawn per replicate and five replicates, following recommendations on pseudo-absence number and design for the algorithms used ([51], Table S2). Both the target-group background and the pseudo-absences were drawn from the accessible area (M), delimited as the union of the native and colonised range polygons buffered by 50 km, following the accessible-area concept [52]. Model projections used Türkiye buffered by 150 km.

Models were fitted in biomod2 v.4.3-4-5 [27,53]. Seven algorithms were used to construct the ensemble. These were generalised linear models, generalised additive models, generalised boosted models, MaxEnt via maxnet, artificial neural networks, classification tree analysis, and multivariate adaptive regression splines. Random forest was included only as the class-balancing down-sampled variant (RFd), because standard random forest tends to perform poorly on imbalanced presence-background data, classifying almost all background as suitable and returning near-zero discrimination [54]. All RFd sub-models nonetheless failed during fitting, because the class-balancing down-sampling size exceeded the number of presences available in each cross-validation partition (a known limitation of the down-sampled random forest routine in the biomod2 version used). RFd therefore produced no usable models and was excluded before the performance-threshold step, and the ensemble was built from the seven algorithms that fitted successfully. The complete algorithm set, the run design (five pseudo-absence sets combined with five replicate 80/20 random splits, giving 25 runs per algorithm), and the basis for excluding random forest are detailed in the Supplementary Material (Section S3). For the MaxEnt model, feature classes and the regularisation multiplier were tuned with ENMeval using the target-group background and spatially blocked cross-validation. The setting that minimised the small-sample corrected Akaike information criterion (AICc) was selected, which favoured linear features with a regularisation multiplier of 5.0 [55,56,57,58]. The remaining six algorithms used the pre-tuned default (‘bigboss’) parameter set in biomod2. Models were calibrated and internally evaluated with five replicate random splits, each using 80% of the data for training and 20% for testing.

An ensemble was built by computing a weighted mean of the individual model predictions, with weights proportional to the True Skill Statistic, and ensembling across pseudo-absence sets and runs [27,59]. Only models meeting a minimum performance threshold (True Skill Statistic ≥ 0.50) contributed to the ensemble, so poorly performing single models did not degrade the consensus prediction. Predictor importance was quantified with the permutation procedure implemented in biomod2 (three permutation rounds per model), averaged across algorithms, pseudo-absence sets, and folds, and accumulated local-effects response curves were computed for each predictor.

Predictive performance was evaluated with both threshold-dependent and threshold-independent metrics. The True Skill Statistic and the area under the receiver operating characteristic curve summarised discrimination [60]. The area under the curve is interpreted with appropriate caution, because for presence-background data it is sensitive to the extent of the background and does not indicate calibration by itself [61]. The continuous Boyce index was therefore relied upon as the primary measure of how well predicted suitability matches the observed frequency of presence, since this metric is designed for presence-only data [62]. Residual spatial autocorrelation was assessed on the ensemble residuals across the calibration extent rather than the Türkiye projection grid, so that the diagnostic reflected the data used for model fitting [63,64]. Residuals were evaluated by presence and background locations (*n* = 24,087) under two neighbourhood definitions. These were a distance band of 0–100 km and k-nearest neighbours with k = 8. As at this sample size the permutation *p*-values are near-deterministically small (the expected value of I approaches zero), the magnitude of I and the shape of the correlogram were used to judge dependence rather than significance alone [65]. The resulting values are reported in the Results and Figure S2. Spatially blocked cross-validation was used when tuning the MaxEnt settings, whereas the ensemble members were evaluated with random data splits, so the cross-validated discrimination scores may be optimistic given this residual autocorrelation. The continuous Boyce index and the magnitude of the residual autocorrelation are therefore relied upon as more cautious checks, and this diagnostic is treated as complementary evidence rather than a pass-or-fail criterion [30]. Spatial statistics were computed with the spdep package in R.

### 2.4. Spatial Projections, Range Change, and Niche Dynamics

The calibrated ensemble was projected onto the current climate of Türkiye and onto future climates for two horizons, 2041–2070 and 2071–2100. Future climate was represented by CHELSA-CMIP6 bioclimatic layers for five global climate models (MPI-ESM1-2-HR, MRI-ESM2-0, IPSL-CM6A-LR, GFDL-ESM4, and UKESM1-0-LL) under the three Shared Socioeconomic Pathways available in that product (SSP1-2.6, SSP3-7.0, and SSP5-8.5) [66]. UKESM1-0-LL has a high equilibrium climate sensitivity, and its projections occupy the warm tail of the model spread. The results are therefore reported across the full model ensemble rather than relying on any single model [67].

To map binary suitable and unsuitable areas, two thresholds derived from the current prediction were applied. These were the thresholds that maximise the sum of sensitivity and specificity (MaxSSS) and the tenth-percentile training-presence threshold (P10) [68,69]. Range change for each future scenario was computed from the binary maps using the maximum sum of sensitivity and specificity threshold, restricted to Türkiye. For each scenario, stable, lost, and gained area in square kilometres, the net change, and the percentage change relative to the current suitable area were recorded [70].

The geometry of projected range change was summarised in two complementary ways. First, the geographic centroid of the suitable area under current and future climate was computed, together with the distance and bearing of centroid displacement, as a measure of the direction and magnitude of range movement [4]. Second, the rate of advance at the leading edge was quantified by regressing the latitudinal position of the northern range margin against time over the observed series, following the leading-edge approach used for climate-driven range shifts [8]. Both a regression-based rate and an endpoint-based rate are reported.

Whether the niche occupied in the colonised range is conserved relative to the native niche was tested using the COUE framework, which partitions niche change into centroid shift, overlap, unfilling, and expansion within a gridded environmental space [16,17]. The environmental space was constructed from a principal component analysis of the five retained predictors across both ranges, and Schoener’s *D* and Warren’s *I* were then computed as measures of niche overlap. Niche equivalency and niche similarity were tested with permutation tests [71], using the intersection of available environments and a 100 × 100 grid resolution with 1000 permutations, following the ecospat framework and its recent applications to niche-overlap tests [72,73]. Niche expansion, stability, and unfilling were quantified on the shared environmental space. The sensitivity of niche stability to the native-versus-colonised sampling asymmetry was assessed by rarefying the native occurrences down to each colonised sample size (*n* = 689 with complete predictor values, and *n* = 23, with 100 replicates each) and recomputing the niche-dynamics indices. Separately, a geometric baseline for stability was generated from random points drawn from the colonised-range background. The native-calibrated niche was also transferred to the colonised range and evaluated against the independent colonised occurrences with the continuous Boyce index and the native tenth-percentile suitability threshold (Figure S3, Table S3). The transfer test is specified in full in the Supplementary Methods (Section S2).

Transferring a model to a future climate can require predicting into conditions not represented during calibration, which reduces reliability. For every future scenario, the extent of non-analogue climate within Türkiye was quantified using the multivariate environmental similarity surface and the type-two extrapolation metric of the extrapolation detection tool [74,75]. The percentage of the projection area that is non-analogue is reported for each scenario, and these values are used to qualify the confidence of the corresponding range-change estimates. 

### 2.5. Geographical Driver Analysis (OPGD)

The drivers of the spatial pattern of suitability were further examined with the optimal-parameter-based geographical detector (OPGD) [76], implemented in the GD package. This quantifies the power of each predictor to explain the spatial heterogeneity of predicted suitability (the q statistic, ranging from 0 to 1) and detects pairwise interactions between predictors. Because the detector’s response variable is the model’s own predicted suitability, its q values decompose the fitted surface rather than provide an independent test of drivers. Continuous predictors were discretised into four to six classes using the natural breaks, quantile, and standard deviation methods, retaining the combination that maximised q for each predictor. Suitability and predictors were sampled at 2500 random points within Türkiye.

### 2.6. Area of Habitat and Monitoring Priority Mapping

To approximate open-habitat availability, the current climatic suitability was further refined following the area-of-habitat approach [77] by excluding densely forested cells unsuitable for an open-habitat, arid-zone species. These were defined as cells where the ESA WorldCover-derived fractional tree cover (obtained with the geodata R package at approximately 30-arc-second resolution and resampled to the model grid) was ≥0.5 [78]. To operationalise the distribution model as field-monitoring guidance for Türkiye, each climatically suitable cell was classified into one of three non-overlapping priority classes. (1) Suitable and recorded, comprising cells with ensemble weighted-mean suitability ≥ MaxSSS threshold that fell within 30 km of at least one confirmed colonised-range occurrence in Türkiye (*n* = 23). (2) Suitable and unfilled, comprising suitable cells more than 30 km from any Türkiye record, representing climatically suitable territory without confirmed detections and therefore the highest immediate monitoring priority. (3) Projected gain (emerging), comprising cells currently below the suitability threshold but projected to exceed it under the intermediate-warming scenario (SSP3-7.0, 2041–2070, GCM ensemble mean), identifying areas where near-term colonisation is anticipated. The 30 km distance threshold is used as a record-neighbourhood aggregation distance rather than a detection radius, because point-count and eBird detection footprints are much smaller [79]. Its influence on the recorded-versus-unfilled classification was examined at neighbourhood distances of 10, 20, 30, and 50 km, with the resulting sensitivity reported in Section 3.6. Cell-to-record distances were computed with the distance function of the terra package. Class areas were derived from the cellSize function of the terra package. The monitoring-priority map is presented in Section 3.6.

## 3. Results

### 3.1. Occurrence Data, Predictors, and Model Performance

After cleaning and spatial thinning, the dataset comprised 19,126 occurrence records, of which 18,432 fell in the native range and 694 in the colonised range (of which 689 had complete predictor values and were used in the niche-dynamics comparison, and 23 were confirmed Türkiye records). The niche-dynamics comparison uses the colonised records with complete predictor values (*n* = 689), spanning the post-native expansion range from the Levant to Anatolia, whereas the monitoring-priority map (Section 3.6) is restricted to confirmed Türkiye occurrences (*n* = 23). Collinearity filtering and the forced retention of the minimum temperature of the coldest month produced a final set of five predictors. These were the mean diurnal temperature range (BIO2), minimum temperature of the coldest month (BIO6), precipitation of the wettest month (BIO13), precipitation of the driest month (BIO14), and precipitation seasonality (BIO15).

The ensemble discriminated occupied from background conditions well. Across all retained single models, the mean cross-validated True Skill Statistic was 0.602 ± 0.030 and the mean area under the curve was 0.866 ± 0.025 (175 models, seven algorithms with five pseudo-absence sets and five cross-validation folds). The continuous Boyce index of the ensemble was 0.818, indicating that the predicted suitability increased monotonically with the observed frequency of presence (Figure S4). Among individual algorithms, classification tree analysis achieved the highest mean True Skill Statistic (0.645), followed by the artificial neural network (0.629) and the generalised boosted model (0.623). The generalised boosted model achieved the highest mean area under the curve (0.892) and the generalised linear model the lowest (0.813) (Table 1 and Table S4, Figure S5). Residual spatial autocorrelation of the ensemble was positive but declined with distance (global Moran’s *I* = 0.548 for the 0–100 km distance band and 0.762 for k = 8 nearest neighbours; correlogram from 0.563 at 12.5 km to 0.270 at 487.5 km, *p* = 0.001, 999 permutations, Figure S2).

### 3.2. Current Predicted Distribution and Key Drivers

**Figure 2:**
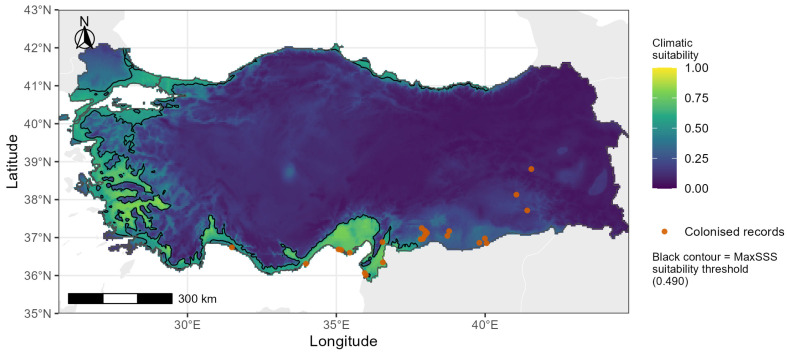
Current climatic suitability. Weighted-mean ensemble (0–1 scale) for *Oena capensis across* Türkiye with colonised-range records overlaid (vermilion). Suitable area at the MaxSSS threshold.

**Figure 3:**
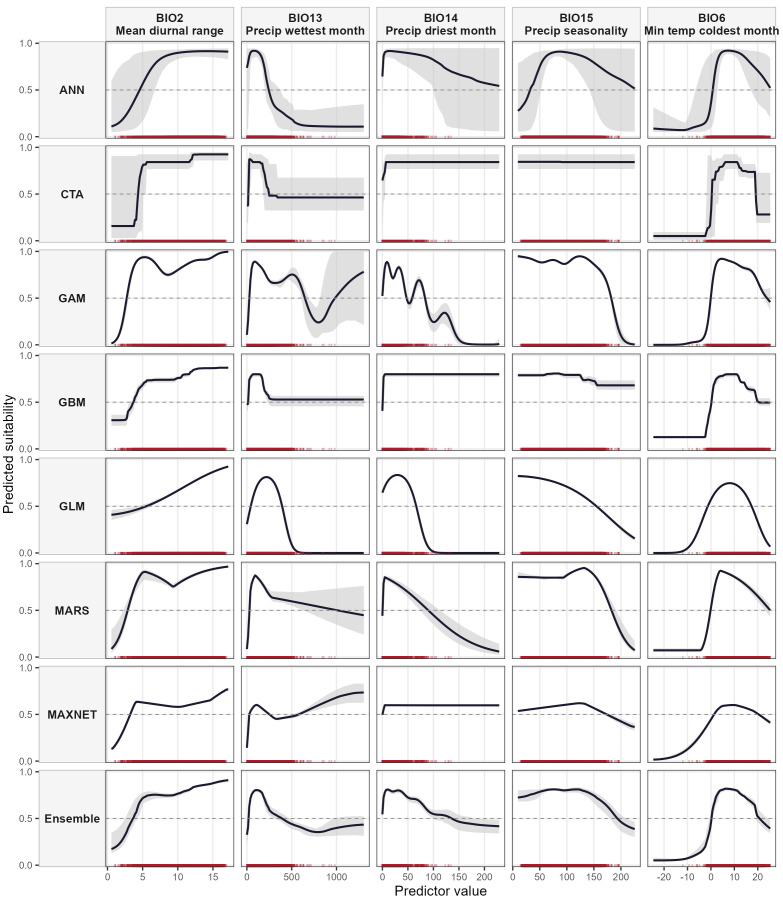
Response curves. Mean predicted suitability (bold line) and the 95% interval (2.5th–97.5th percentile across cross-validated models, band) for the five predictors (x-axes in native units, with °C for BIO2 and BIO6, mm for BIO13 and BIO14, coefficient of variation for BIO15) per algorithm and pooled ensemble. The red rug marks observed values.

Precipitation variables dominated the ensemble. The mean variable importance was highest for the precipitation of the wettest month (0.385), followed by the precipitation of the driest month (0.331) and the minimum temperature of the coldest month (0.279). The mean diurnal range (0.227) and precipitation seasonality (0.158) contributed the least (Figure S6, Table S5). The current suitable area in Türkiye, mapped at the maximum sum of sensitivity and specificity threshold (0.490, tenth-percentile threshold 0.155) (Table S6), was approximately 91,699 km^2^, or about 11.7% of the land area, and was concentrated in the south and west, and parts of the centre, of the country (Figure 2 and Figure S7). The minimum temperature of the coldest month, although retained on physiological grounds and ranked third, contributed appreciably, which is consistent with the role of the low winter temperature at the northern margin (Figure 3 and Figure S8).

### 3.3. Niche Dynamics

**Figure 4:**
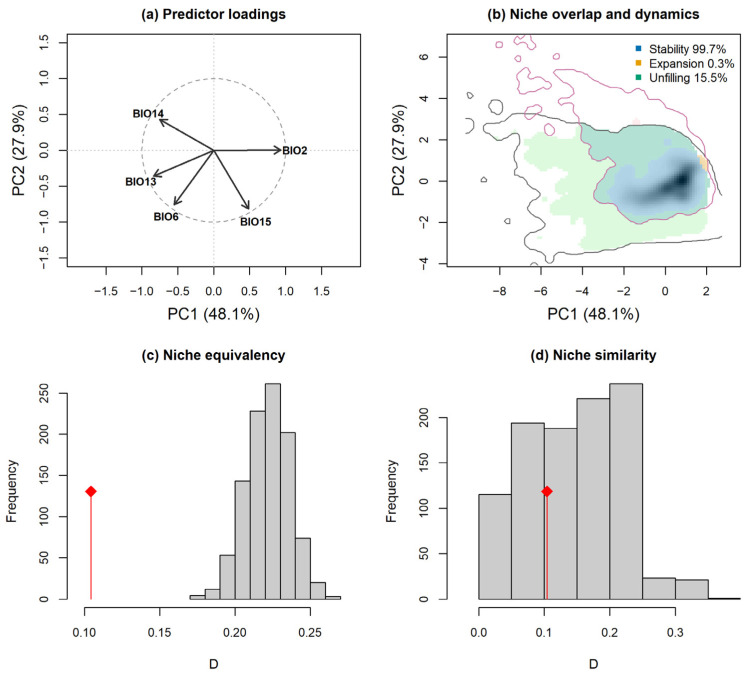
Niche dynamics (COUE framework) for the native and colonised niches of *Oena capensis* in principal-component space. (**a**) Predictor loadings on the first two components, with the percentage of variance explained. (**b**) Niche overlap, with stability (blue), expansion (orange), and unfilling (green), native- and colonised-range occurrence densities shaded, the centroid shift arrowed, and solid and dashed contours delimiting 100% and 50% of the available environment. (**c**) Niche-equivalency and (**d**) niche-similarity null distributions, with the observed Schoener’s D and its significance.

The colonised niche was a contained subset of the native niche, though several lines of evidence indicate that this reflects an early, non-equilibrium expanding front rather than equilibrium conservatism. The niche overlap was low under both metrics (Schoener’s *D* = 0.104, Warren’s *I* = 0.284). The niche equivalency hypothesis was rejected (*p* = 0.001, the minimum attainable with 1000 permutations), indicating that the two niches are not identical. The niche similarity tests were not significant in either direction (native to colonised *p* = 0.686, colonised to native *p* = 0.073), so no evidence was found that the niches are more similar than expected by chance, given the available environments. The equivalency and similarity conclusions were unchanged under Warren’s *I* (Table S7). Within the shared environmental space, niche stability was 99.7%, niche expansion was 0.3%, and niche unfilling was 15.5% (Figure 4, Table S7). Stability and expansion partition the colonised niche and sum to 100%, whereas unfilling is measured against the native niche, so the three indices do not need to add up to 100%. The combination of high stability with negligible expansion indicates that the colonised population occupies conditions already present in the native niche, while the moderate unfilling indicates that part of the native niche remains unoccupied in the colonised range.

Three additional analyses qualified this result (Figure S3, Table S3). First, niche stability was insensitive to the native-to-colonised sampling asymmetry. Rarefying the native occurrences to the colonised sample size left the stability essentially unchanged (0.999 at *n* = 689, 0.948 at *n* = 23), so the contained-subset signal is not an artefact of the larger native sample. Second, stability was near its ceiling in this setting, because random points drawn from the colonised-range background yielded a stability of approximately 0.992. The observed 0.997 therefore exceeded a geometric null only marginally, indicating that stability alone is weak evidence of conservatism. Third, a transfer of the native-calibrated niche to the colonised range predicted the colonised occurrences poorly. The negative continuous Boyce index (−0.65, versus +0.79 for the native self-fit) indicates that native-predicted suitability was inversely, not merely weakly, associated with colonised presence. Although 99.7% of colonised occurrences fell inside the native niche envelope and 89% exceeded the native tenth-percentile suitability, they occupied lower native suitability than the colonised-range background (median 0.057 versus 0.106). This envelope-inclusion percentage is distinct from the COUE stability index, which coincidentally also rounds to 99.7%. The colonised population thus occupies the cool, low-suitability margin of the native niche rather than its optimum, consistent with a still-establishing expanding front.

### 3.4. Future Range Projections and Range-Shift Geometry

**Figure 5:**
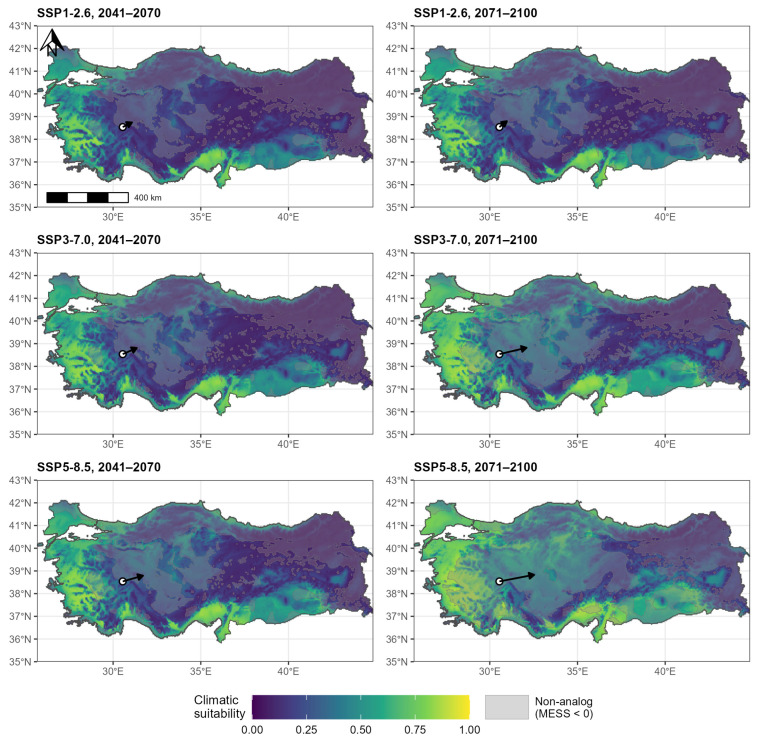
Future climatic suitability. Ensemble projections for *Oena capensis* across Türkiye under three SSPs and two periods (five-GCM mean). Non-analogue climate (MESS < 0) shown as a legended grey overlay, the centroid shift arrowed, and a scale bar and north arrow on each panel.

The suitable area in Türkiye was projected to increase under every scenario, horizon, and model combination examined (30 combinations in total, Table 2, Figure 5). The mean percentage change in the suitable area, averaged across the five global climate models, rose with emissions and time. Under SSP1-2.6, it was +59.0% ± 42.3 for 2041–2070 and +90.0% ± 96.7 for 2071–2100. Under SSP3-7.0, it was +120.2% ± 91.8 and +213.5% ± 147.6, and under SSP5-8.5, it was +151.5% ± 130.0 and +271.8% ± 148.7 (mean ± standard deviation across models). Across all combinations, the percentage change ranged from +4.1% to +519.9% (Figure S9, Table S8), and the projected loss of currently suitable area was small in every case (0 to approximately 4969 km^2^). Between-model variation was large, as the standard deviations indicate, with UKESM1-0-LL consistently producing the most extensive gains (Figure S10).

**Figure 6:**
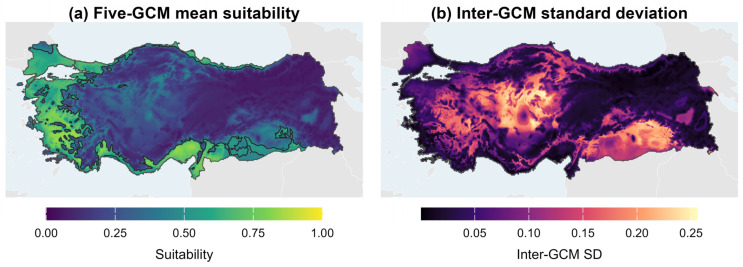
Multi-model uncertainty in the SSP3-7.0, 2041–2070 ensemble projection for *Oena capensis* across Türkiye. (**a**) Five-GCM mean climatic suitability (0–1, grey contour = MaxSSS threshold). (**b**) Inter-GCM standard deviation of suitability. Brighter tones mark greater disagreement among the five global climate models. Perceptually uniform, colourblind-safe palettes (viridis, magma) are used. Türkiye-centred Lambert azimuthal equal-area projection.

The reliability of these projections is constrained by extrapolation. Inter-model spread in the projected suitability was largest over the central and south-eastern interior and smallest in the west (Figure 6). The fraction of the Türkiye projection area classified as a non-analogue climate was high in every scenario, ranging from 70.7% to 95.2% with a mean of approximately 77.6% (Table S9, Figures S11 and S12). The most extreme expansion estimates coincided with the highest non-analogue fractions, so the upper range of projected gains corresponds to the climates least represented in the calibration data and should be interpreted with caution. The non-analogue signal was not dominated by any single predictor but was led by the driest-month precipitation, cold-month minimum temperature, and precipitation seasonality (Figure S13).

**Figure 7:**
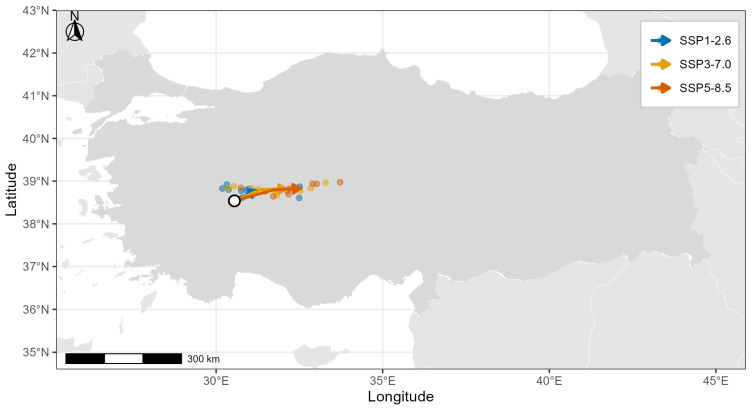
Centroid trajectories of climatically suitable area for *Oena capensis* in Türkiye under future climate. Arrows connect the current-climate suitability centroid (open circle) to the ensemble-mean centroid for each SSP scenario and horizon. Faint coloured points show all 30 scenarios, horizons, and climate-model combinations. The arrows show a predominantly eastward and north-eastward, rather than purely poleward, displacement.

Projected range shifts were predominantly eastward rather than purely poleward. Centroid displacement of the suitable area ranged from approximately 21 to 280 km, with a mean of approximately 107 km, and increased with emissions and time. The mean displacement bearing was approximately 54°, and 23 of 30 combinations fell within an east–northeast to east–southeast band (bearings of 45° to 135°), indicating an eastward component to the projected movement (Figure 5 and Figure 7). The largest displacement, approximately 280 km on a bearing of about 79°, occurred under SSP5-8.5 for 2071–2100 with UKESM1-0-LL. 

At the leading edge, the regression of the northern margin position against time over the observed 12-year series yielded a positive slope of approximately 2.44 km yr^−1^, while the endpoint-based rate was approximately −0.40 km yr^−1^. These are reported as descriptive rates. The two disagree because the endpoints are unrepresentative of the intervening records, so neither is treated as a tested velocity. The statistical significance of the leading-edge regression was not established in the present analysis, and the rate is not interpreted as a tested trend (Table S10).

### 3.5. Geographical Drivers of Climatic Suitability

**Figure 8:**
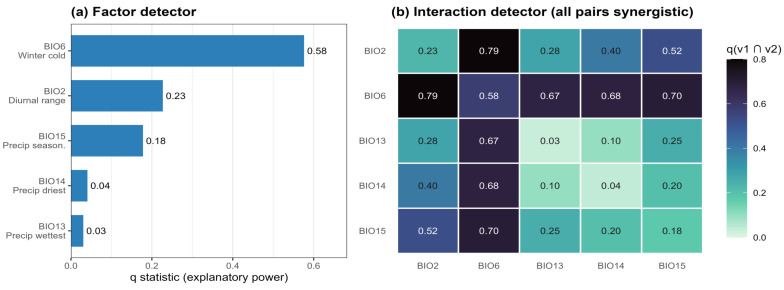
Driving-factor analysis. Optimal parameters geographical detector. (**a**) Factor-detector q (power to explain spatial heterogeneity). (**b**) Interaction detector (joint q, all pairs synergistic).

In the geographical-detector analysis, the minimum temperature of the coldest month had the highest power to explain the spatial pattern of suitability within Türkiye (q = 0.576), followed by the mean diurnal range (q = 0.227) and precipitation seasonality (q = 0.178). The precipitation of the wettest and driest months had low explanatory power (q = 0.03 and 0.04, respectively). All predictor pairs interacted synergistically (enhancement), with the strongest being the mean diurnal range with the minimum temperature of the coldest month (q = 0.795). Every pair involving a low winter temperature exceeded q = 0.67 (Figure 8, Table 3). The full interaction matrix is given in Table S11.

### 3.6. Monitoring Priority and Habitat Refinement

**Figure 9:**
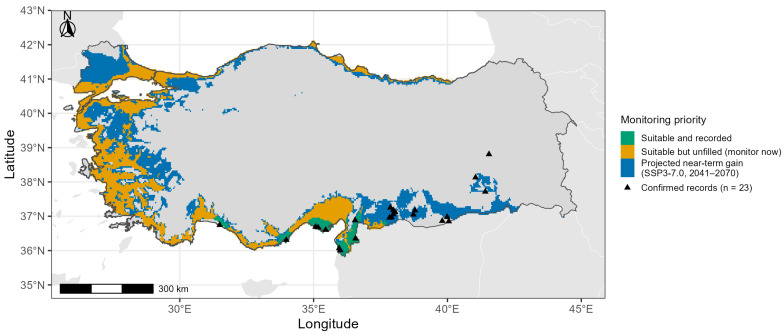
Monitoring priority map. Climatically suitable cells (MaxSSS threshold) are classified as suitable and recorded (within 30 km of a confirmed Türkiye occurrence), suitable but unfilled (more than 30 km from any record, the highest immediate monitoring priority), or projected near-term gain (below the current threshold but suitable under SSP3-7.0, 2041–2070) for *Oena capensis* in Türkiye. The 30 km is a record-neighbourhood aggregation distance (Section 2.6). Confirmed occurrences are marked with triangles, and a colourblind-safe categorical palette is used.

Of the 91,699 km^2^ currently classified as climatically suitable in Türkiye, approximately 9108 km^2^ (~10%) fell within 30 km of a confirmed occurrence record (suitable and recorded class), while the remaining 82,591 km^2^ (~90%) constituted suitable but unfilled territory with no record within the 30 km record neighbourhood distance (Figure 9). Under SSP3-7.0 2041–2070, a further 93,755 km^2^ is projected to enter the suitable range as an emerging gain class (identified on the GCM ensemble mean suitability surface, so it is not directly comparable with the threshold-then-averaged gains in Table 2), concentrated in central Anatolia and the eastern interior. The unfilled suitable area exceeds the recorded area roughly nine-fold. This unfilled fraction probably reflects incomplete community-science coverage, dispersal lag at an expanding front, and unmodelled land-cover and biotic constraints, not detection failure alone. 

Refining climatic suitability by open-habitat availability (the area-of-habitat approach [77]) reduced the suitable area from 91,699 to 61,855 km^2^. Of the climatically suitable land, 32.5% (29,844 km^2^) fell in dense forest unsuitable for this open-habitat dove, concentrated on the southern Taurus (~13,939 km^2^) and northern Black Sea (~4960 km^2^) coasts (Figure S14, Table S12). The recorded-versus-unfilled monitoring classification was likewise insensitive to the choice of neighbourhood distance, with the unfilled fraction decreasing only from 98% at 10 km to 82% at 50 km (Table S13).

## 4. Discussion

The climatic suitability of Türkiye for *Oena capensis* was modelled and projected under the future climate, and the niche dynamics of its northward expansion were tested. The climate of southern and central Türkiye is already suitable for the species. The suitable area is projected to increase under every scenario and model combination, with negligible loss, although most of the projected future climate is non-analogue. The colonised population occupies a contained subset of the native niche, with high stability and negligible expansion. Together, these results indicate that *O. capensis* is expanding along the cool margin of the native niche rather than establishing in a novel climatic space.

### 4.1. Climatic Drivers of Suitability

Two complementary analyses identified different leading variables, and the distinction matters for interpretation. Across the species’ whole range, the ensemble permutation importance was dominated by precipitation, with the precipitation of the wettest and driest months being the two most important predictors and the precipitation seasonality contributing less (Figure 3), consistent with the arid-zone affinity of the species. Within Türkiye, by contrast, the geographical detector identified the minimum temperature of the coldest month as the strongest correlate of the spatial pattern of suitability (q = 0.576, Section 3.5). These results are not contradictory. Permutation importance describes what defines the niche across the native and colonised ranges, whereas the detector describes what structures suitability along the specific climatic gradients of Anatolia, where winter cold sets the northern margin. The synergistic interaction between the minimum winter temperature and the mean diurnal range (joint q = 0.795) further indicates that suitability along the Anatolian margin is structured jointly by winter cold and its diurnal variability (continentality) rather than by either alone, consistent with a cold continental interior limit. Because the detector decomposes the model’s own surface, this interaction is descriptive rather than an independent mechanism. Because no species-specific thermal-tolerance data are available for *Oena capensis*, this winter-margin mechanism is advanced as an a priori hypothesis for testing rather than as an established physiological limit. The first hypothesis (that precipitation and low winter temperatures limit suitability) is therefore supported. The species tolerates extreme heat through efficient evaporative cooling, remaining thermally stable at air temperatures well above 50 °C [19]. Water availability, expressed through precipitation of the wettest and driest months (a proxy for the seasonal availability of seeds and sparse herbaceous cover in open arid landscapes), is therefore a more likely arid-margin constraint than heat stress. Where the range instead abuts cool climates, as at the Anatolian margin, the thermal tolerance is expected to govern how far the leading edge advances [80]. The minimum winter temperature, through frost exposure and reduced cold-season resources, may then set the northern limit that warming would relax. Summer heat extremes, drought-driven seed failure, and vegetation change could impose limits at the warm, dry end of the gradient, so this winter-cold relaxation may not translate into a net benefit. These Anatolian conditions, though warm and dry within Türkiye, correspond to the cooler, drier margin of the species’ native African and Arabian niche rather than its warm core. This correspondence between Anatolian climate and the native niche’s cool margin is quantified in the niche dynamics analysis (Section 4.2, Figure S3).

### 4.2. Niche Dynamics and Comparison with Other Expansions

The third hypothesis (that the colonised niche would remain largely within the native niche) was only partly supported. The niche-dynamics results are most consistent with partial filling of the native niche rather than expansion into novel climate, but they fall short of demonstrating full niche conservatism. On the supporting side, niche expansion was negligible (0.3%) and the contained-subset signal held under native-sample rarefaction. On the cautionary side, the absolute overlap was low (Schoener’s *D* = 0.104, Warren’s *I* = 0.284), and the similarity tests were non-significant in both directions [81]. In addition, observed stability (99.7%) barely exceeded a random-placement (geometric) baseline of about 0.992, and a native-calibrated model transferred poorly to the colonised range (continuous Boyce index −0.65). The low overlap and high stability are not in themselves contradictory. Schoener’s *D* compares occupancy density across the whole shared environmental space, whereas stability measures the share of the colonised niche that falls within native-occupied conditions, so a colonising population occupying a small but contained subset of the native niche yields low D with high stability. Because that high stability is close to a random-placement baseline, however, the contained-subset inference rests on stability being insensitive to native-sample rarefaction rather than on its absolute value. The unfilling of 15.5% indicates that some native-niche conditions remain unoccupied at the expanding margin, with the expected signature of a range still filling rather than being at equilibrium. Following Strubbe et al. [15], observed niche differences in non-native birds often reflect partial filling of the native niche rather than true niche change, and Guisan et al. [17] caution that overlap metrics must be read against environmental availability and analogy. The most defensible conclusion is therefore deliberately narrow. The colonists currently occupy a contained, cool, low-suitability margin of the native niche, consistent with a non-equilibrium expanding front rather than with equilibrium conservatism. The 15.5% unfilling is modest relative to the higher unfilling typically reported for non-native European birds [15] and, unlike the expansion-dominated signal recovered for established European breeders, is consistent with an earlier, still-filling front. Two cautions apply. First, the colonised sample pools a longer-established, near-equilibrium population in the Levant and Arabia with the actively expanding Anatolian front, so the contained-subset signal may reflect this mixture rather than the dynamics of the front alone. The rarefaction test addresses sample-size asymmetry but not this equilibrium heterogeneity. Second, low overlap together with a failed native-to-colonised transfer is not by itself diagnostic of partial niche filling, because the same signal can arise from early-stage colonisation, uneven propagule pressure, biassed occurrence recording, and the limited accessible area of a still-spreading front [52,82]. The contained-subset interpretation is therefore offered as a cautious, within-sample interpretation that remains subordinate to the more decisive out-of-sample transfer test, which was not passed. Poor reciprocal transferability of this kind is itself an expected signature of niche estimation under non-equilibrium and argues for recalibrating on combined native and colonised data as colonisation proceeds [82]. The unfilling, the high non-analogue fraction, and the extensive suitable-but-unoccupied area are not separate findings but one coherent non-equilibrium signal [83]. A realised niche that is a contained subset of the fundamental niche is also anticipated on Hutchinsonian grounds where competing or otherwise limiting taxa are present [84], linking the present pattern to the columbid-interaction hypotheses developed below (Section 4.6).

This contained-subset pattern is consistent with the broader literature on the conservatism of climatic niches during animal invasions and expansions [17,81]. A similar pattern of niche conservatism has been reported across many invasive species, which largely conserve their native climatic niche during establishment [85]. The same signature was observed in invasive alien ants, which show smaller climatic-niche shifts than non-invasive congeners [38], and in invasive parakeets, which retain much of their native climatic niche when colonising Europe [15]. Niche conservatism is a widely supported organising principle that supports the assumption that a model calibrated on the native range can inform suitability elsewhere [14]. The present results extend the test of this pattern to an Afrotropical and Saharo-Arabian columbid expanding into the Western Palearctic, a system in which formal niche-overlap tests had not previously been applied. Niche conservatism is not universal, however. Climatic-niche shifts have been documented during some avian invasions and range expansions: for example, in species with greater behavioural flexibility, or where colonisation history and local climate velocities differ [86,87,88,89]. Indeed, a comparable ensemble and niche-overlap framework applied to a rapidly expanding waterbird recovered pronounced niche expansion rather than conservatism [90]. The niche pattern recovered here therefore characterises this expansion rather than being assumed a priori. Its contained-subset component is supported by a niche stability that is insensitive to native-sample rarefaction and by negligible expansion, whereas its non-equilibrium character is shown by the failed native-to-colonised transfer and the cool-margin position of the colonists, not by the modelling framework itself. A native-calibrated model transferred to the colonised range is the more decisive test of conservatism [91], and that test was not passed here (Section 3.3).

### 4.3. Direction and Rate of Range Change

As the fourth hypothesis anticipated, the projected movement of suitable areas was predominantly eastward rather than poleward. This non-poleward pattern is consistent with evidence that climate-driven range shifts frequently depart from a simple poleward expectation because they track the multivariate climate, including precipitation, over complex topography [4,5]. The centroid displacement summarised here is a geometric property of the projected suitability surface rather than a measured dispersal vector. Its mean bearing (about 54°) is better attributed to the spatial arrangement of suitable climate and land cover across Anatolia than to any intrinsic directional preference. That arrangement forms an aridity and winter-temperature corridor of mild-wintered, open dry lowlands and agricultural mosaics rather than a latitudinal axis. In the Eurasian Collared Dove, an apparently innate directional spread was likewise shown to be largely explained by the geometry of the suitable habitat and climate [92]. The eastward signal is unlikely to be a detection artefact, because range-shift records are geometrically biassed toward latitudinal rather than longitudinal movement, so longitudinal shifts tend to be under-recorded [93]. Because the reporting intensity is nonetheless spatially uneven, the eastward signal should be re-tested against observer coverage as records accumulate. More broadly, syntheses increasingly find that observed range shifts are multidirectional rather than uniformly poleward, reflecting local climate-change velocities and multivariate climate gradients [94,95,96]. European breeding birds, for instance, have shifted north and north-eastwards rather than due north [89], and a recent northward range expansion of a passerine in Asia provides a further parallel [97]. A comparable climate-driven expansion of an arid-affiliated bird into interior Anatolia has recently been documented for the Cream-coloured Courser (*Cursorius cursor*) [98], lending a regional, same-biome analogue to the eastward range shift inferred here. In Türkiye, where strong west–east and coastal–interior climatic gradients are imposed by topography, an eastward shift in a precipitation-limited niche is a plausible geographic expression of climate tracking. Consistent with a strong climatic structuring of the Turkish avifauna, country-wide bird species richness differs significantly among geographic regions and Köppen–Geiger climate classes [99].

The leading-edge analysis must be interpreted conservatively. The regression-based rate of approximately 2.44 km yr^−1^ over a 12-year window is a descriptive summary of the observed northern margin, and the endpoint-based rate was slightly negative, which reflects year-to-year variation in a short and expanding series. The short series, the small colonised sample, and the non-equilibrium nature of an ongoing expansion all caution against treating the rate as a tested velocity. It is therefore presented as a preliminary descriptive estimate that should be revisited as more records accumulate. Records at the expanding margin are consistent with the pattern in which vagrant individuals form the vanguard of climate-driven range shifts, preceding establishment [100,101,102]. The failed native-model transfer and the low native suitability of the colonised records (Figure S3) quantitatively corroborate this non-equilibrium, vanguard pattern, which complements the climate-tracking interpretation. Breeding has been reported in Anatolia [23] and is well established immediately to the south and east [22], indicating that current Türkiye records reflect an expansion front that has begun to establish rather than a settled range. In the Eurasian Collared Dove, another expanding columbid, the North American invasion front has been found to advance faster across less-suitable, peripheral niche conditions than across the most-suitable niche core, a pattern attributed to longer-distance dispersal where intervening areas are unfavourable [103]. This pattern indicates that the rate and direction of an expanding margin need not track the most climatically suitable areas directly. In the same species, dispersal flights along a coastal barrier were strongly directional and aligned with the invasion vector, indicating that the direction of spread can be structured rather than random [104]. 

More broadly, the pace and geometry of columbid range expansion are often governed by non-climatic factors. These include dispersal behaviour that can advance fronts across less climatically suitable space [103,104] and anthropogenic resource creation, such as large irrigation schemes (for example, the Southeastern Anatolia Project), which can reshape granivore distributions independently of bioclimate [105]. The realised geography of any advance is, moreover, likely to track dispersal corridors (river valleys, irrigated plains, and urban and agricultural mosaics) more closely than the raw bioclimatic surface, because the most successful columbid colonists are village and farmland commensals. Anthropogenic water and cultivation may thus decouple the realised resource availability from the macroclimate, and irrigated oases concentrate resources into stepping-stone patches that can scaffold spread across otherwise hostile arid gaps [106,107]. Climatic suitability is therefore a permissive background rather than a sufficient explanation for the observed spread. Independently of the present model, a country-wide analysis of Turkish community-science data recovered a significant positive occupancy trend for the species between 2001 and 2022 [23], corroborating that O. capensis is increasing at the Palearctic margin even though the leading-edge rate estimated here remains inconclusive. Published continental rates of avian range-margin advance provide context but are not directly comparable, because they are estimated over different periods, regions, and methods [2,8].

### 4.4. Non-Analogue Climate and the Interpretation of Projected Gains

The most consequential caveat concerns non-analogue climate. Across scenarios, the projection area classified as non-analogue averaged about 78% and rose to roughly 85% under the highest-emission, end-of-century scenario, exceeding 95% in the most extreme individual combinations. Predictions into non-analogue space are extrapolations, and, as emphasised for niche-shift inference, apparent gains in such space may reflect climates that were simply unavailable during calibration rather than a well-characterised biological response [17,74,75]. The largest projected gains coincided with the highest non-analogue fractions and the widest spread among climate models, so the upper end of the range-change estimates is also the least reliable. The second hypothesis (that suitable area increases under warming) was supported. Accordingly, the direction of the response, a net gain in suitable area, is the best-supported result, because it is mechanistically plausible for a warm-adapted, arid-zone species and holds across every scenario and model. The magnitude is not. The multi-hundred-percent gains projected under high-emission, end-of-century, high-sensitivity combinations should be read as scenario illustrations conditioned on a non-analogue climate, not as calibrated forecasts of future area. Reporting the non-analogue fraction alongside each projection, as done here, allows the reliability of each estimate to be judged directly, in line with current standards for transparent niche-model transfer [32,108]. This combination of a presence-only-appropriate evaluation metric (the continuous Boyce index), an explicit non-analogue quantification, and a formal niche-overlap test provides a more cautious basis for inference than reliance on a single discrimination statistic alone. More broadly, the use of correlative models to forecast the expansion of a spreading species is itself debated. Such forecasts are most defensible where extrapolation is limited and the niche is conserved, conditions met only in part here, given both the high non-analogue fraction and the failed native-to-colonised niche transfer [109,110]. Because these projections extend largely into the non-analogue climate, even the directional inference is not immune to extrapolation and should be re-tested as records and climate data accumulate.

The projected gain in suitable area for *O. capensis* also contrasts informatively with recent niche-model studies of resident Turkish taxa. Climate projections for the brown bear in Türkiye indicate the contraction and upslope displacement of the suitable habitat under warming, the response expected of a cool-adapted, montane species [33]. Assessments of breeding vultures and of a freshwater turtle in the region similarly emphasise scenario-dependent redistribution of the suitable area [34,35]. Even a range-expanding bird in the same country, the introduced Rose-ringed Parakeet, was projected to lose about half of its suitable Turkish habitat by 2100 [111], a warm-affiliated colonist nonetheless facing contraction. Warm affiliation alone does not fix the direction of response. However, an earlier niche model of Turkish songbirds projected several warm-affiliated residents to expand under warming, with the White-spectacled Bulbul gaining the most suitable area and shifting from southern toward northern Anatolia [12]. Closer ecological analogues point the same way. Within the same region, the Laughing Dove (*Spilopelia senegalensis*), an arid-origin columbid, is expanding across southern Türkiye as a year-round breeder, implying high reproductive output as an engine of range gain [112]. Together with the Cream-coloured Courser (Section 4.3), these expansions indicate that arid-zone birds are entering Anatolia alongside *O. capensis* rather than in isolation. More broadly, ensemble niche models have documented climate-driven range shifts and niche dynamics for many regional taxa [113,114,115,116]. The present results place *O. capensis* at this expanding end of the spectrum, a warm-adapted colonist for which the same regional warming that contracts cool-adapted residents expands suitability. Climate change therefore does not move all range margins in the same direction, and the sign of the response depends on the species’ thermal and hydric niche [1,4].

### 4.5. Limitations

This study has several limitations. First, the model is correlative and Grinnellian. It estimates abiotic suitability from the climate and ignores biotic interactions, dispersal, and microhabitats, so the projected suitability is potential rather than realised occupancy, and it tends to over-predict occupied area [17]. Consistent with this, an area-of-habitat refinement [77] identified about 32.5% of the climatically suitable area in dense forest as being unsuitable for an open-habitat dove (Figure S14). This over-prediction is systematic rather than regional, consistent with the accessible-area framing of niche models [52], and adding land cover or an aridity index would reduce it. Removing forested cells corrects one habitat mismatch but does not establish that the remaining area is ecologically available, which also depends on cultivation, bare ground, grazing, settlement structure, and water access. Second, presence-only data carry residual sampling bias despite target-group background and spatial thinning, which can skew suitability toward well-surveyed areas, though such models have proved reliable for the related European Turtle Dove [117]. Third, the colonised range is sampled during an active, non-equilibrium expansion. Niche stability was insensitive to the native-to-colonised sampling asymmetry (Table S3), but the small colonised sample (689 records) still widens the uncertainty of the niche-similarity tests and the leading-edge estimate. Because breeding in Türkiye, though reported [23], is recent and localised, and some peripheral records may be of vagrant or captive origin [21,22] (a provenance confound well documented for traded cage-birds [118]), the colonised signal probably reflects an early-stage, still-establishing population. Fourth, the minimum temperature of the coldest month was imposed a priori on physiological grounds, so its ranked importance is inflated relative to a purely data-driven ordering. Fifth, because the ensemble was evaluated with random rather than spatially blocked splits, the cross-validated scores are likely optimistic and the effective sample size is smaller than the nominal record count [30,119]. The full spatial diagnostic is reported for transparency [64], and environmental filtering indicated only a few hundred unique predictor combinations [120]. Independent validation would require temporally or spatially independent records, which were unavailable [121]. Finally, and most consequentially, the high non-analogue fraction means the long-term, high-emission projections extrapolate well beyond the calibration climate, constraining confidence in the magnitude, though not the direction, of projected gains. Adding vegetation structure as a predictor or habitat mask is recommended for future models. These limitations bias the results in known directions. The climate-only model, the high non-analogue fraction, and the random-split cross-validation each tend to make suitability and its projected gains optimistic, whereas removing peripheral vagrants narrows the colonised sample and can inflate estimated niche stability. The directional conclusions are therefore more secure than the area estimates.

### 4.6. Conservation Relevance and Future Work

These results have practical value for anticipating colonisation at the Western Palearctic margin. A contained-subset, precipitation-dominated niche and projected expansion under warming together identify where monitoring is most likely to detect range filling. In practice, monitoring could combine model-guided stratified point counts or transects in the suitable-but-unfilled and projected-gain zones [122] with structured contributions to national atlas and community-science schemes [123] analysed under current best-practice guidelines for community-science data [79]. Adding mechanistic layers of physiological tolerance would help delimit winter-survival limits and the species’ dependence on irrigated agricultural landscapes [124]. To make this operational, the monitoring-priority map (Figure 9) separates climatically suitable but as-yet unfilled areas, where established colonisation could be detected now, from areas of projected near-term gain (SSP3-7.0, 2041–2070) in the interior and east, where surveillance should expand. Because roughly 90% of the currently suitable area (about 82,600 km^2^) remains unfilled, more than 30 km from any record, the scope for detecting ongoing establishment is large, while the open-habitat refinement (Figure S14) narrows attention to non-forested terrain. As records accumulate, they should be incorporated to strengthen the leading-edge and niche-similarity estimates, and adding land cover, habitat structure, and, where data permit, physiological tolerances would move projections from potential toward realised suitability and reduce reliance on non-analogue extrapolation.

Although *O. capensis* is not itself a species of conservation concern, its arrival as a columbid colonist in Anatolia raises the question of how it might interact with resident species, including native doves and other columbids. Two classes of interaction are relevant where range-expanding or introduced columbids reach high local densities. The first is competition with resident granivores. The Eurasian Collared Dove (*Streptopelia decaocto*), the most studied columbid colonist, expanded rapidly across Europe and North America in human-modified, grain-rich landscapes [125], yet clear competitive effects on native doves have not been demonstrated even after decades of co-occurrence [126,127]. Within Türkiye, the Laughing Dove has expanded and now breeds year-round in the south, where a concurrent decline in *S. decaocto* has been read as possible competitive displacement [112]. The second is the role of columbids as reservoirs of *Trichomonas gallinae*, which is prevalent in wild columbids near the study region [128,129]. The same parasite caused epidemic mortality and population declines in wild passerines, most severely in European Greenfinches (*Chloris chloris*) and, to a lesser extent, Common Chaffinches (*Fringilla coelebs*) [130,131], and cross-species transmission is favoured where columbids and passerines share provisioned food and water. Pathogen emergence has likewise accompanied other columbid range expansions [132]. For *O. capensis*, which occurs at low density and with only recent, localised breeding in Türkiye [23], neither interaction has yet been proven. Both are raised as hypotheses for future surveillance, which could be complemented by attention to local granivore assemblages and, where birds congregate at water, to parasite prevalence. These interactions yield testable, directional predictions. Resource competition should matter most where *Oena capensis* reaches high density in grain-rich, human-modified habitats shared with resident *Streptopelia* and *Columba* doves, and can be tested as spatial co-occurrence or local exclusion against habitat quality. For Trichomonas gallinae, the relevant risk is disease-mediated apparent competition, with prevalence at shared water points being the natural monitoring indicator, since a colonist entering assemblages that already circulate the parasite could acquire and amplify shared strains.

## 5. Conclusions

Ensemble niche modelling shows that southern and central Türkiye is already climatically suitable for *Oena capensis*. The suitable area is projected to increase under every emission scenario examined, with negligible loss. In environmental space, the colonised records occupy the cool, low-suitability margin of the native niche and only partly fill it. This pattern is consistent with an expanding front that tracks the suitable climate rather than shifting into novel conditions. Because most of the projected long-term climate has no present-day analogue, the direction of change is better supported than its magnitude. The projected gain is a net expansion, mainly eastward. This eastward bias reflects the geometry of suitable climate across Anatolia rather than an intrinsic direction. The niche inference remains cautious and testable rather than settled. The study provides a reproducible, transparently documented baseline for anticipating and monitoring this Afrotropical columbid at the Western Palearctic margin. It can be refined as records accumulate and land-cover and mechanistic data are added.

## Figures and Tables

**Table 1:** Predictive performance of the individual algorithms and the weighted-mean ensemble for *Oena capensis* (cross-validation means ± standard deviation). TSS, True Skill Statistic; AUC, area under the receiver operating characteristic curve; CBI, continuous Boyce index (ensemble only).

Algorithm	*n*	TSS Mean	TSS SD	AUC Mean	AUC SD	Boyce CBI
CTA	25	0.645	0.01	0.881	0.007	NA
ANN	25	0.629	0.011	0.886	0.007	NA
GBM	25	0.623	0.01	0.892	0.004	NA
MARS	25	0.593	0.009	0.867	0.005	NA
GAM	25	0.586	0.009	0.868	0.004	NA
GLM	25	0.58	0.011	0.813	0.006	NA
MAXNET	25	0.561	0.01	0.854	0.004	NA
Ensemble (EMwmean)	175	NA	NA	NA	NA	0.818

**Table 2:** Projected change in climatically suitable area for *Oena capensis* across Türkiye under three SSP scenarios and two periods, averaged across five GCMs. Analogue-only gain restricts the gain to cells with analogue climate (MESS ≥ 0). Areas are thresholded per GCM then averaged, so values differ slightly (<3%) from Table S8.

Scenario	Period	Current (km^2^)	Future (km^2^)	Gain (km^2^)	Analog-Only Gain (km^2^)	Loss (km^2^, Mean; Max)	Change (%)	Non-Analogue (%)
SSP1-2.6	2041–2070	91,699	146,000 ± 39,000	55,000 ± 38,000	33,000 ± 23,000	989; 2587	+59.0 ± 42.3	74.6
SSP1-2.6	2071–2100	91,699	174,000 ± 89,000	84,000 ± 88,000	45,000 ± 40,000	1297; 4969	+90.0 ± 96.7	76.1
SSP3-7.0	2041–2070	91,699	202,000 ± 84,000	111,000 ± 85,000	64,000 ± 40,000	457; 1706	+120.2 ± 91.8	74.6
SSP3-7.0	2071–2100	91,699	287,000 ± 135,000	197,000 ± 136,000	86,000 ± 37,000	1043; 2276	+213.5 ± 147.6	78.7
SSP5-8.5	2041–2070	91,699	231,000 ± 119,000	139,000 ± 120,000	66,000 ± 39,000	544; 1810	+151.5 ± 130.0	76.6
SSP5-8.5	2071–2100	91,699	341,000 ± 136,000	250,000 ± 137,000	88,000 ± 63,000	1140; 3031	+271.8 ± 148.7	85.1

**Table 3:** Driving factors of climatic suitability within Türkiye (optimal parameters geographical detector, factor detector). q is the power to explain the spatial heterogeneity of predicted suitability; pairwise interaction q-values are given in Table S11 and Figure 8b.

Predictor	Description	q	*p*
BIO6	Minimum temperature of the coldest month	0.576	<0.001
BIO2	Mean diurnal range	0.227	<0.001
BIO15	Precipitation seasonality	0.178	<0.001
BIO14	Precipitation of the driest month	0.041	<0.001
BIO13	Precipitation of the wettest month	0.031	<0.001

## Data Availability

The species occurrence records retrieved from the Global Biodiversity Information Facility are available as a GBIF Occurrence Download. Available online: https://doi.org/10.15468/dl.7787j8 (accessed on 13 June 2026). Additional georeferenced occurrences were obtained from the regional literature cited in the text. CHELSA version 2.1 and CHELSA-CMIP6 climate layers are publicly available from their respective providers. Analysis code and derived results tables are available from the author upon reasonable request.

## Supplementary Materials

The following supporting information can be downloaded at: https://www.mdpi.com/article/10.3390/ani16142238/s1. Figure S1: Predictor correlation; Figure S2: Residual autocorrelation; Figure S3: Sensitivity analyses of the niche-conservatism inference; Figure S4: Boyce index; Figure S5: Algorithm performance; Figure S6: Predictor importance; Figure S7: Prediction uncertainty; Figure S8: ALE response curves; Figure S9: BBinary range change; Figure S10: Inter-GCM spread; Figure S11: MESS surface; Figure S12: ExDet NT2 surface; Figure S13: Predictor driving climatic novelty (most dissimilar variable) for the SSP3-7.0, 2041–2070 projection; Figure S14: AOH refinement; Table S1: ODMAP protocol; Table S2: Sample size and prevalence; Table S3: Sensitivity of niche stability to native-sample rarefaction and native-to-colonised transfer metrics; Table S4: Per-algorithm sensitivity, specificity, TSS, and AUC; Table S5: Predictors retained and their ensemble importance; Table S6: Threshold-choice sensitivity of the current suitable area; Table S7: Niche-dynamics metrics; Table S8: Gain, loss, and stable suitable area; Table S9: Non-analogue climate fraction per scenario; Table S10: Leading-edge expansion; Table S11: OPGD interaction matrix (values shown in Figure 8b); Table S12: Area-of-habitat sensitivity to forest-cover threshold; Table S13: Sensitivity of the monitoring classification to the record-neighbourhood aggregation distance.

## Abbreviations

ALE	accumulated local effects
AUC	area under the receiver operating characteristic curve
BIO2/BIO6/BIO13/BIO14/BIO15	bioclimatic variables
CBI	continuous Boyce index
COUE	centroid-overlap unfilling-expansion niche-dynamics framework
ENM	ecological niche model
ExDet	extrapolation detection
GCM	global climate model
M	accessible area
MaxSSS	maximum of sensitivity plus specificity
MESS	multivariate environmental similarity surface
NT1/NT2	univariate and combinatorial novelty indices of ExDet
ODMAP	Overview data model assessment prediction
P10	10th-percentile training-presence threshold
PCA	principal component analysis
SDM	species distribution model
SSP	shared socioeconomic pathway
TSS	True Skill Statistic
VIF	variance inflation factor
