# Supplementary material for: Ensemble Niche Modelling Projects Net Suitability Gain and Eastward Range Expansion for the Namaqua Dove (Oena capensis) in Anatolia Under Climate Change
Source: Animals (Basel). 2026 Jul 19;16(14):2238. doi: 10.3390/ani16142238 (PMC13403669; doi:10.3390/ani16142238)
Supplement: Supplementary file 1 [file animals-16-02238-s001.zip › animals-4441889-supplementary.pdf]

Supplementary Materials

Ensemble Niche Modelling Projects Net Suitability Gain and Eastward Range Expansion for the Namaqua Dove (*Oena capensis*) in Anatolia under Climate Change

Bekir KABASAKAL

Supplementary Methods

S1. Native and colonised range classification

Each thinned occurrence record was assigned to the native or the colonised (expanding) range by a rule that combined a country-level test with expert-set spatial overrides based on published range accounts [1,2]. First, a record was classified as native if it fell within the dissolved polygon union of the native-range countries (sub-Saharan Africa, Madagascar, and south-western Arabia). All remaining records were provisionally colonised. Second, bounding-box overrides adjusted this where national borders cut across the range edge. South-western Arabia (34–45.5° E, 16–25° N, and 53–60° E, 16–20° N) and the Gebel Elba massif were reclassified as native, whereas the Egyptian Nile and Delta (25–37° E, 22–32° N) were treated as being colonised. Third, records at the southern-African and Senegalese margins misplaced by coastline resolution were reclassified as native by their coordinates. Records isolated from the contiguous northward expansion front were treated as vagrants and excluded. These comprised north-west European, Iberian and Greek, western-Indian (Gujarat), central-Asian, and Australasian records, and, within Türkiye, records north of 40° N, west of 30° E, or on the central-Anatolian steppe (31–35° E, north of 38° N), all of which lie outside the documented Levant-to-Anatolia colonisation axis. The intervening Türkiye records for 2007–2011 are compiled in the national bird report [3]. After spatial thinning at 5 km, the retained set comprised 18,432 native and 694 colonised records (689 of the colonised records had complete predictor values and 23 were in Türkiye, Table S2). These spatial thresholds are expert judgements grounded in the cited range accounts rather than an algorithmic distance criterion. The complete, deterministic assignment is reproducible from the analysis code.

S2. Native-to-colonised niche transfer test

Niche conservatism was additionally assessed by transferring the native niche to the colonised range in environmental space (principal-component space of the retained predictors) [4], rather than as a geographic projection. The availability-corrected native occurrence-density surface from the COUE ordination (the kernel of the native occurrences over the pooled native-and-colonised background principal-component space) was queried at the environmental scores of the colonised occurrences and of the colonised-range background. Evaluated against the colonised occurrences, the continuous Boyce index [5] of this native surface was −0.65 (bootstrap 95% confidence interval −0.81 to −0.38), against +0.79 for the native self-fit and −0.08 for a random-placement baseline. The negative value indicates that native-predicted suitability was inversely associated with colonised presence. The tenth percentile of native suitability evaluated at the native occurrences was used as an inclusion threshold. Of the colonised occurrences, 89% met or exceeded it and 99.7% fell within the native envelope, yet their median native suitability (0.057) was lower than that of the colonised-range background (0.106). This out-of-sample transfer is distinct from, and more decisive than, the within-sample stability index. Because its Boyce index is negative, it argues against full niche conservatism (Section 4.2).

S3. Model algorithms, run design, and the exclusion of random forest

Eight algorithms were initially specified for the biomod2 ensemble: namely, generalised linear models, generalised additive models, generalised boosted models, a down-sampled random forest (RFd), MaxEnt via maxnet, artificial neural networks, classification tree analysis, and multivariate adaptive regression splines. Standard random forest was not used, because on imbalanced presence-background data, it tends to classify almost all background as suitable and to return near-zero discrimination [6], so the class-balancing down-sampled variant (RFd) was specified in its place. Each algorithm was calibrated and internally evaluated over 25 runs, formed by combining five pseudo-absence sets with five-fold cross-validation (80% training and 20% testing per fold). All 25 RFd sub-models failed during fitting, because the class-balancing down-sampling size exceeded the number of presences available in each cross-validation partition, a known limitation of the down-sampled random forest routine in the biomod2 version used [6,7]. RFd therefore produced no usable models and was not retried, so a single, consistent model set was preserved across all downstream steps. The ensemble was consequently built from the seven algorithms that fitted successfully, each represented by its 25 runs (175 model runs in total, Table S4 and Figure S5).

Model selection and screening involved several further steps. Predictors were screened for multicollinearity with the variance inflation factor procedure, retaining only variables below the conventional threshold of 10, and the minimum temperature of the coldest month was kept a priori on physiological grounds. For the MaxEnt model, feature classes and the regularisation multiplier were tuned with ENMeval under spatially blocked cross-validation, selecting the configuration that minimised the small-sample corrected Akaike information criterion. Only individual models meeting a minimum performance threshold (True Skill Statistic greater than or equal to 0.50) contributed to the

weighted-mean ensemble, so that poorly performing single models did not degrade the consensus prediction. Pseudo-absences were drawn as five sets of 10,000 from the accessible area, and the continuous Boyce index was used as the primary, presence-only-appropriate measure of predictive performance.

Supplementary References

1. Biricik, M.; Karakaş, R.; Turğa, Ş. The Namaqua Dove *Oena capensis* Spreads Further North: A New Record from Turkey. *Acta Zool. Bulg.* **2018**, *70*, 35-38.
2. Ławicki, Ł. Go north – range extension of Namaqua Dove in the Palearctic and South Asia. *Dutch Birding* **2020**, *42*, 103-111.
3. Kirwan, G.M.; Özen, M.; Ertuhan, M.; Atahan, A. Turkey Bird Report 2007-2011. *Sandgrouse* **2014**, *36*, 146-175.
4. Broennimann, O.; Fitzpatrick, M.C.; Pearman, P.B.; Petitpierre, B.; Pellissier, L.; Yoccoz, N.G.; Thuiller, W.; Fortin, M.; Randin, C.; Zimmermann, N.E.; et al. Measuring ecological niche overlap from occurrence and spatial environmental data. *Glob. Ecol. Biogeogr.* **2012**, *21*, 481-497. https://doi.org/10.1111/j.1466-8238.2011.00698.x
5. Hirzel, A.H.; Le Lay, G.; Helfer, V.; Randin, C.; Guisan, A. Evaluating the ability of habitat suitability models to predict species presences. *Ecol. Modell.* **2006**, *199*, 142-152. https://doi.org/10.1016/j.ecolmodel.2006.05.017
6. Valavi, R.; Guillera-Arroita, G.; Lahoz-Monfort, J.J.; Elith, J. Predictive performance of presence-only species distribution models: a benchmark study with reproducible code. *Ecol. Monogr.* **2022**, *92*, e01486. https://doi.org/10.1002/ecm.1486
7. Guéguen, M.; Blancheteau, H.; Lemaire-Patin, R.; Thuiller, W. *biomod2: Ensemble Platform for Species Distribution Modeling, R package version 4.3; 2025. https://doi.org/10.32614/cran.package.biomod2*

Supplementary Figures

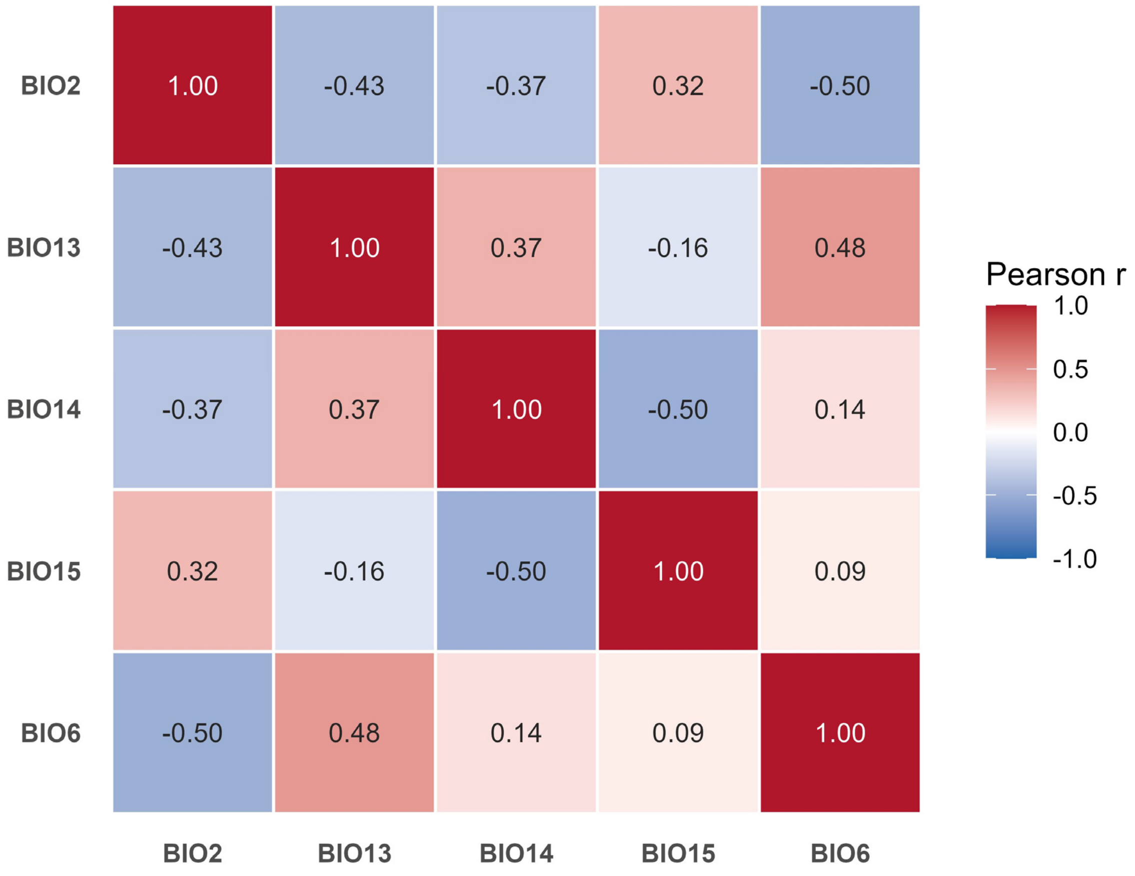

Figure S1. Predictor correlation. Pearson correlation matrix among the five retained predictors, computed over the calibration area (M). The correlations among the precipitation predictors motivate the accumulated-local-effects response curves (Figure S8), which stay interpretable when predictors are correlated.

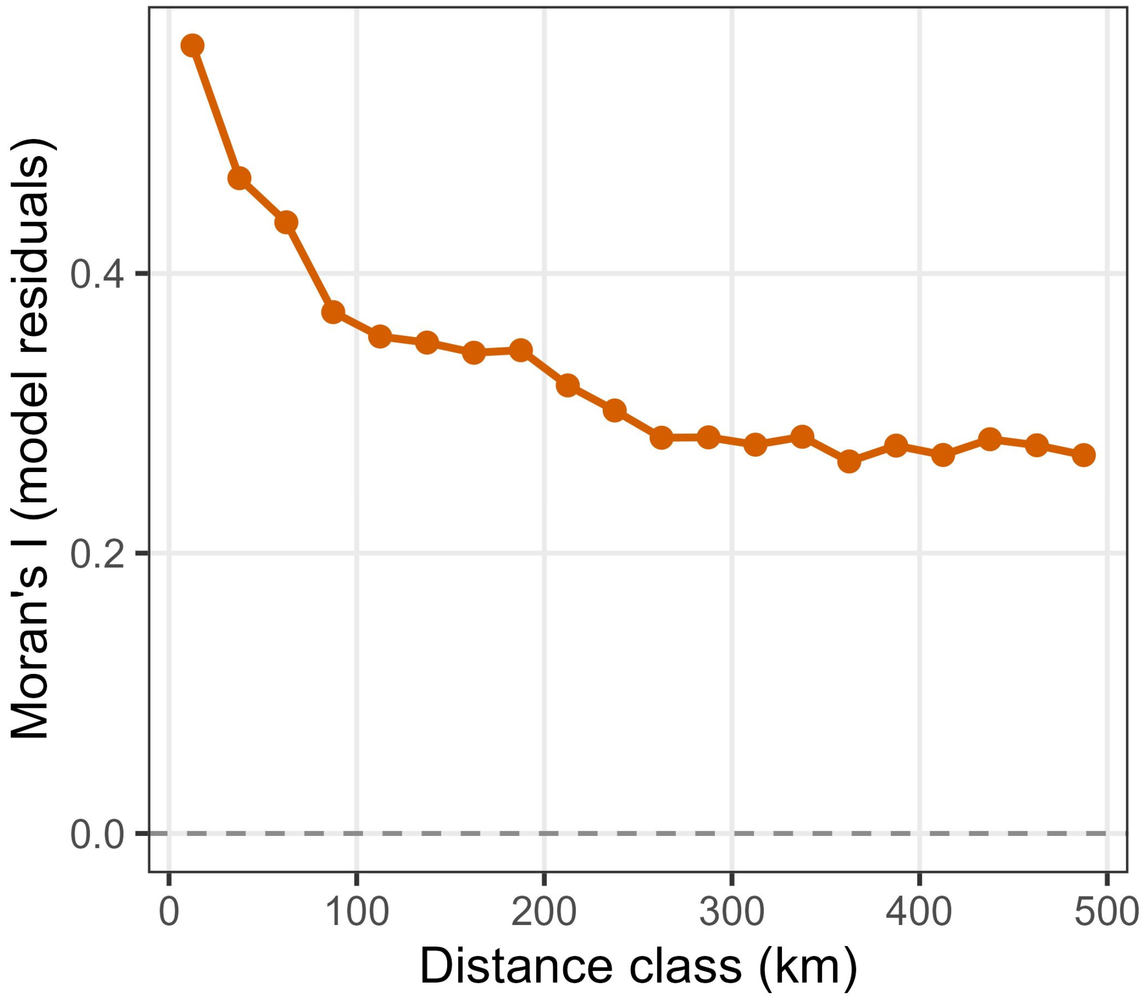

Figure S2. Residual autocorrelation. Moran's I correlogram of the ensemble residuals across distance classes, computed over the calibration extent. Residual autocorrelation declined with distance. The spatially structured residuals are acknowledged in the limitations.

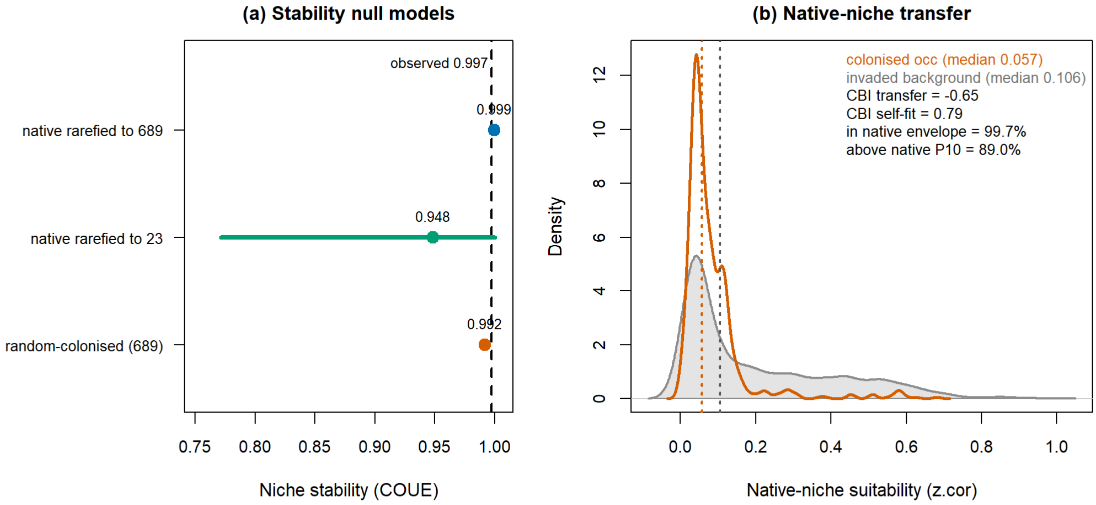

Figure S3. Sensitivity analyses of the niche-conservatism inference. (a) Niche stability under three null models, native occurrences rarefied to the colonised sample size (689 and 23) and random points from the colonised-range background (689), with the observed stability marked by the dashed line. (b) Transfer of the native-calibrated niche

to the colonised range, native-niche suitability at colonised occurrences (vermilion) versus the colonised-range background (grey). Dashed lines mark medians. Values are reported in Table S3.

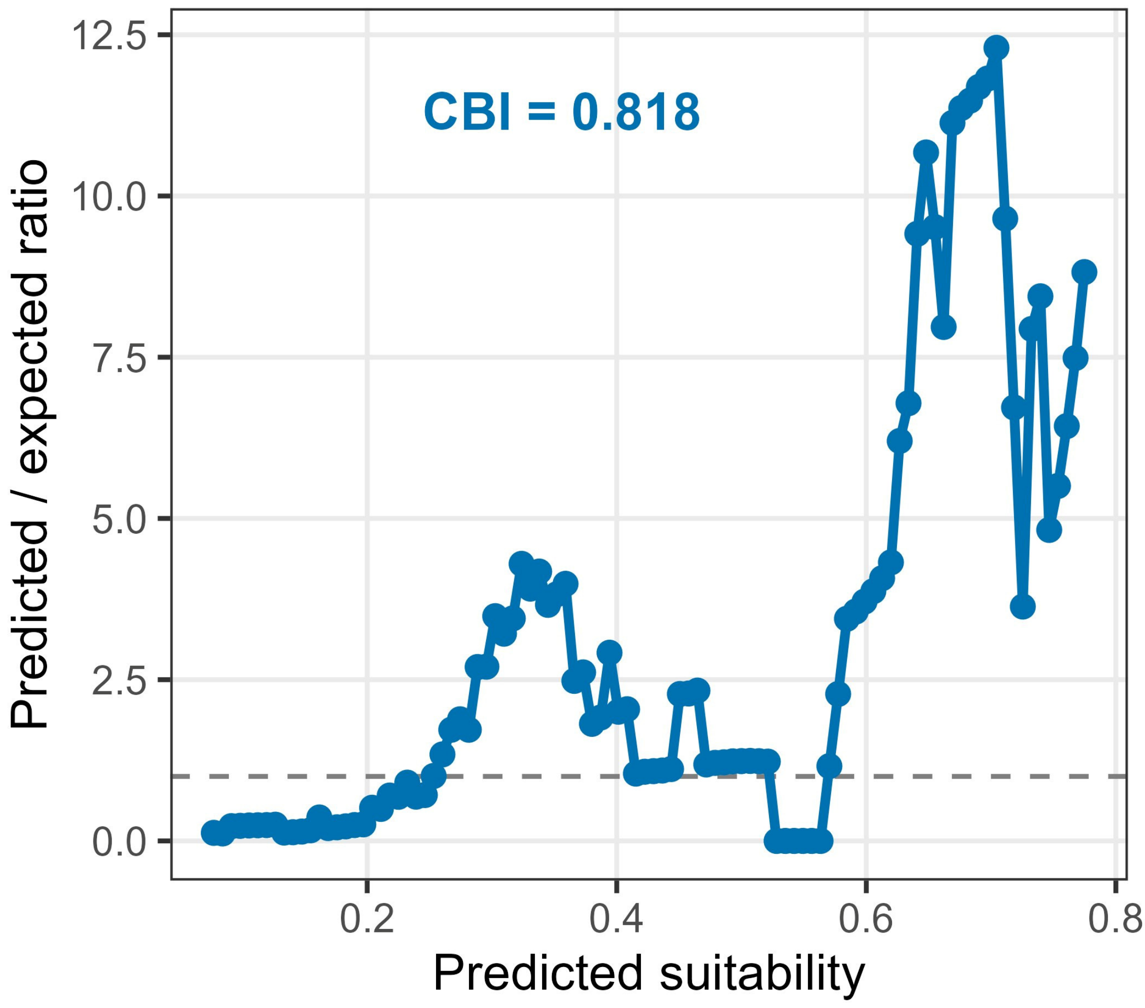

Figure S4. Boyce index. Continuous Boyce index for the current ensemble, shown as the predicted-to-expected ratio across binned suitability. Values above the dashed reference line of 1 indicate that suitable classes were occupied more than expected by chance.

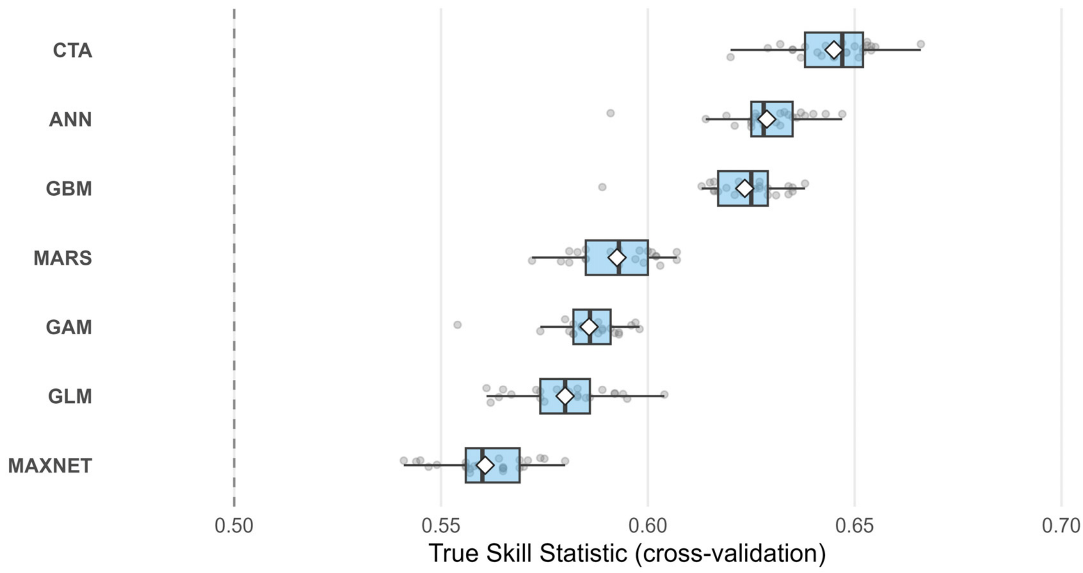

Figure S5. Algorithm performance. True Skill Statistic (TSS) from five-fold cross-validation for each algorithm, shown as a boxplot with jittered folds and a white-diamond mean. The dashed line marks TSS = 0.5. The seven retained algorithms are shown. Random forest failed to fit and was excluded.

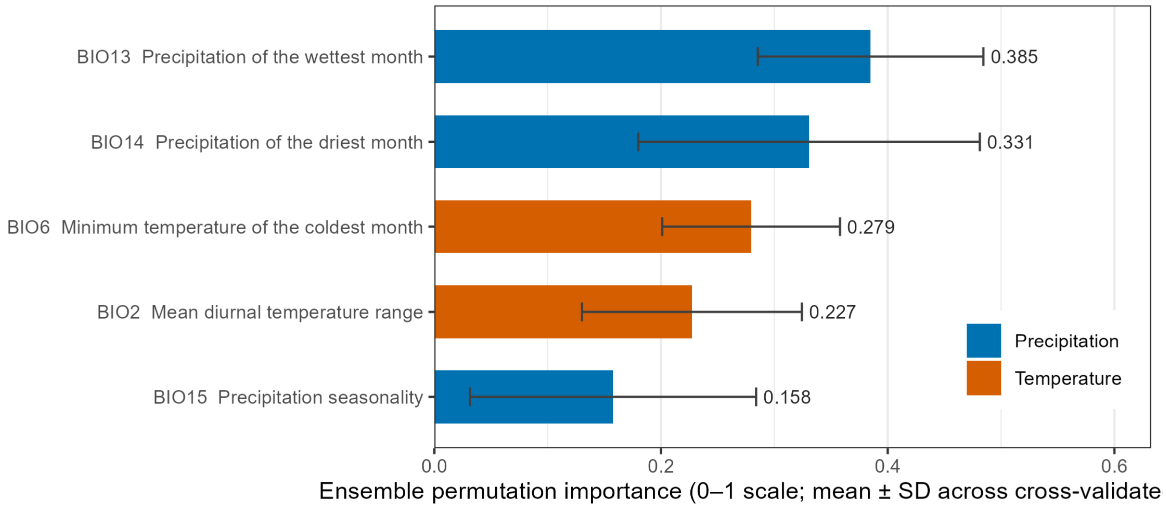

Figure S6. Predictor importance. Ensemble permutation importance (mean and standard deviation across cross-validated models) of the five predictors retained for *Oena capensis*. The values and ranking are reported in Table S5. BIO6 was retained a priori as a northern cold-tolerance constraint.

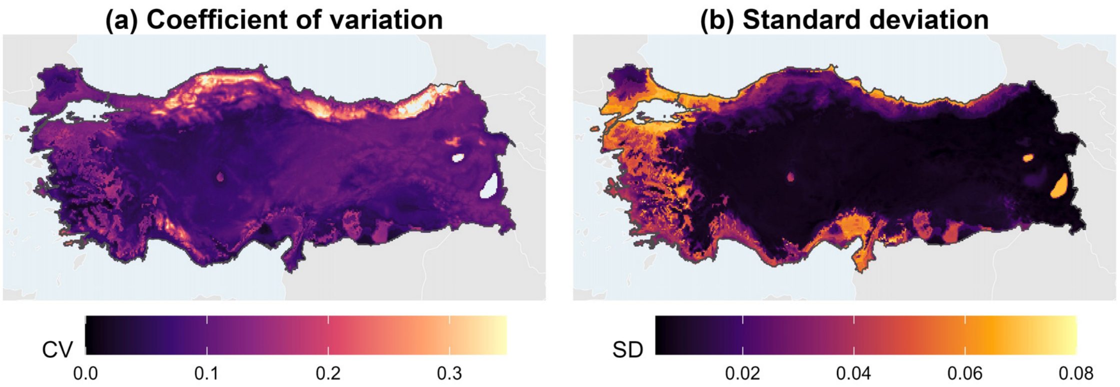

Figure S7. Prediction uncertainty. Uncertainty of the current ensemble prediction across Türkiye, shown as (a) the coefficient of variation and (b) the standard deviation among ensemble-member predictions. Uncertainty was highest at the margins of the predicted suitable area.

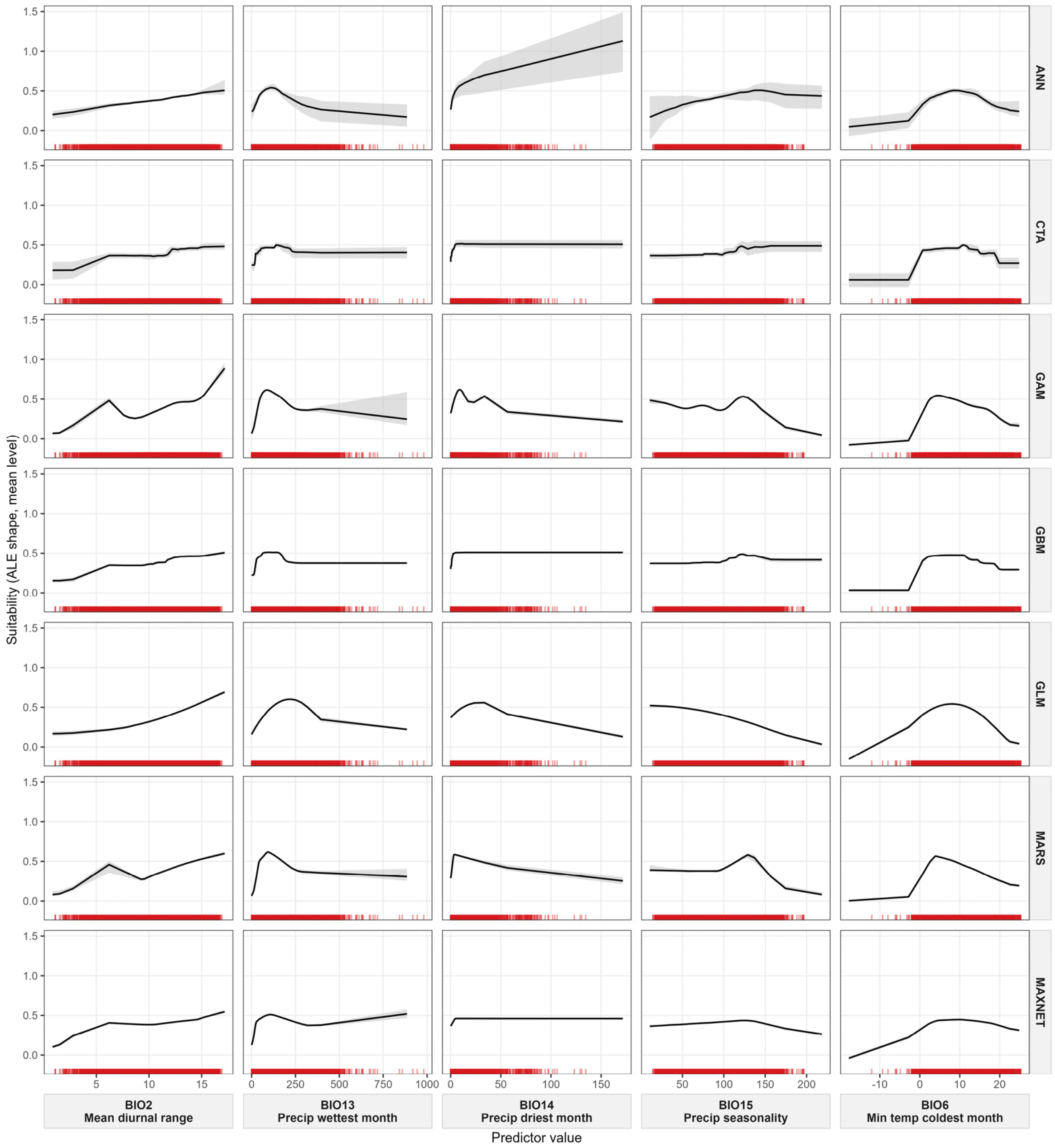

Figure S8. ALE response curves. Per-algorithm accumulated-local-effects (ALE) curves for the five predictors (algorithms in rows, predictors in columns). The line is the mean ALE across cross-validated models, the band is the 5th–95th percentile, and the red rug is the observed values. ALE is insensitive to correlation among the predictors.

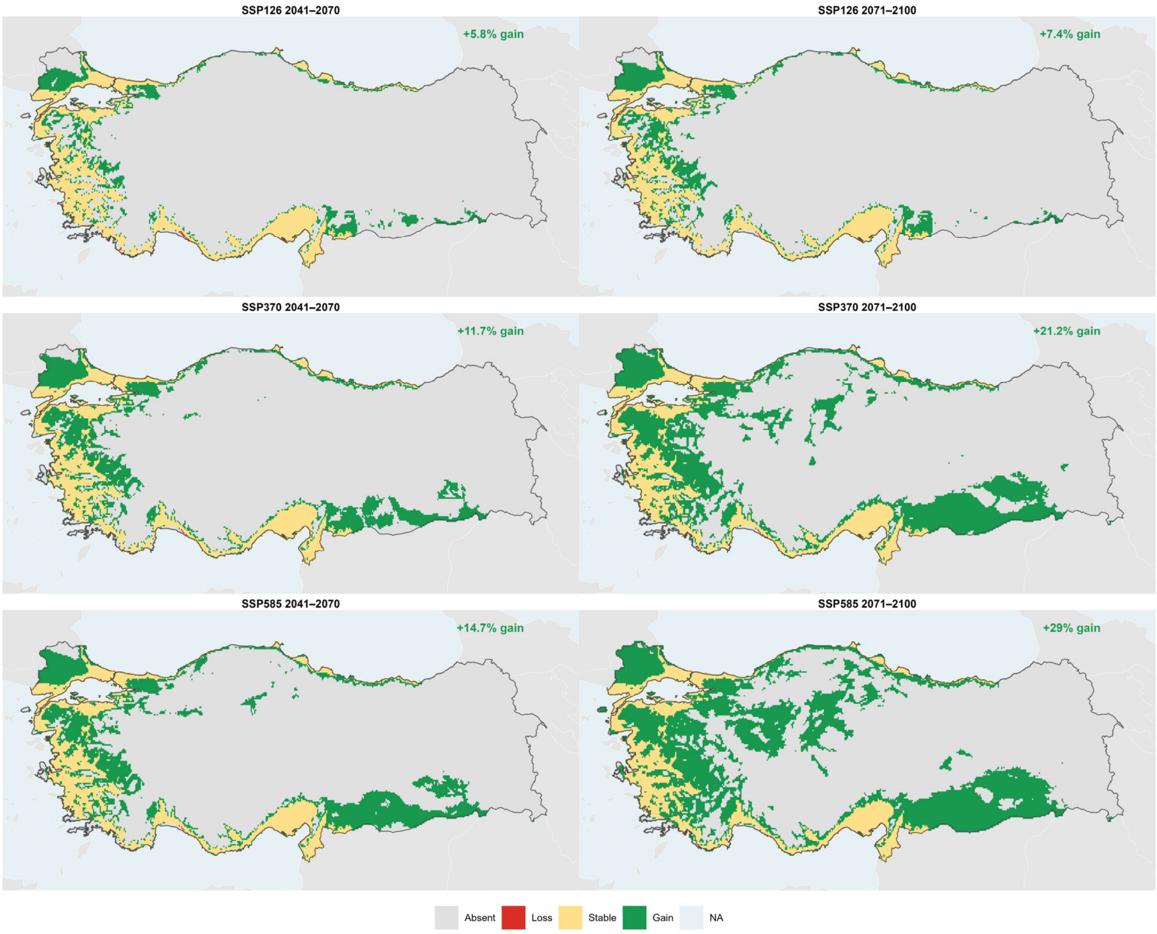

Figure S9. Binary range change. Cells classified as stable, gained, or absent for *Oena capensis* under three SSPs and two periods, by applying the MaxSSS threshold (0.490) to the current and GCM-averaged future ensembles. The percentage gained is annotated on each panel. Projected loss was negligible.

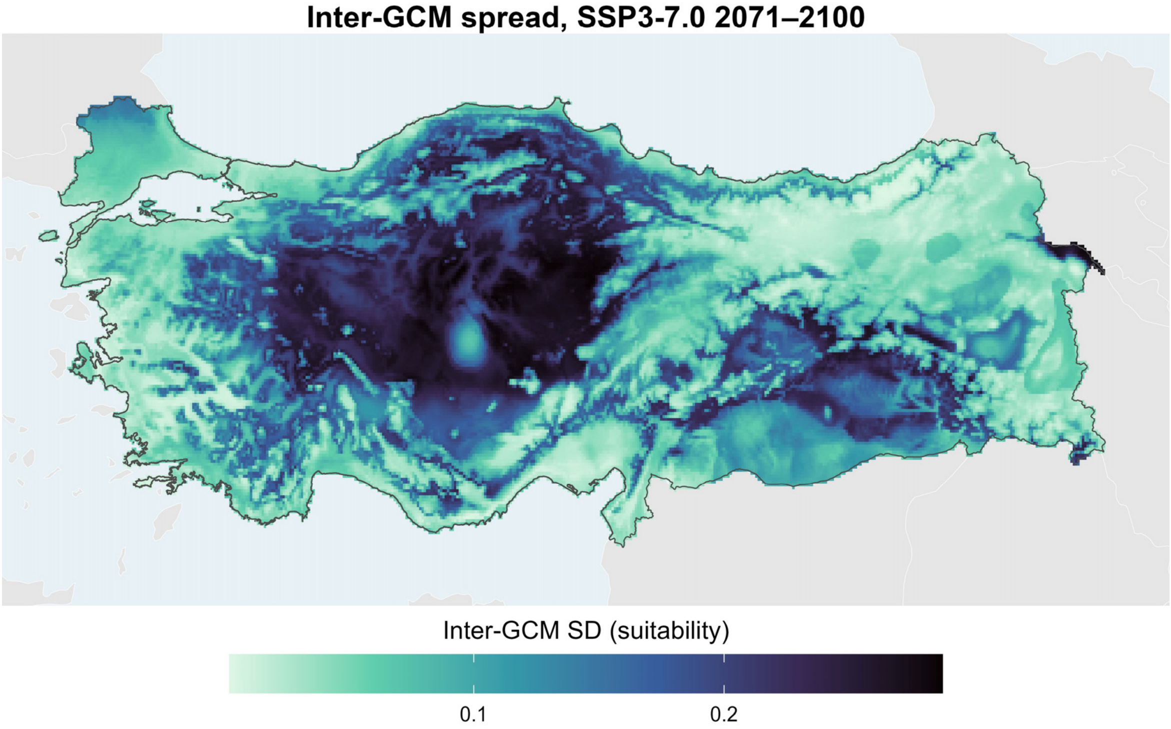

Figure S10. Inter-GCM spread. Per-cell standard deviation of the projected suitability for *Oena capensis* across the five GCMs under SSP3-7.0 for 2071–2100. Larger values mark areas where the projection is most sensitive to the choice of climate model.

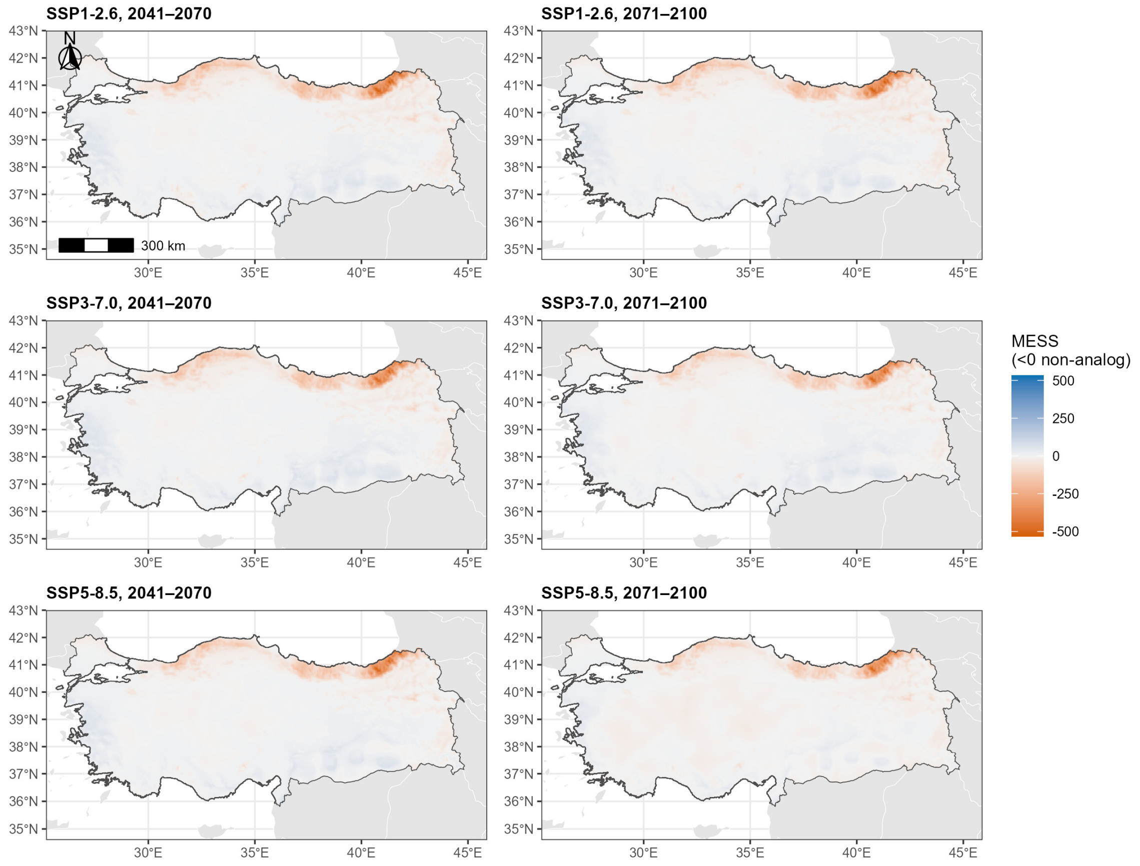

Figure S11. MESS. Multivariate environmental similarity surface (MESS) for each scenario-period combination, averaged across the five GCMs. Negative values (red) denote the non-analogue climate outside the calibration range. MESS expresses univariate (NT1) novelty, complemented by the NT2 surface in Figure S12.

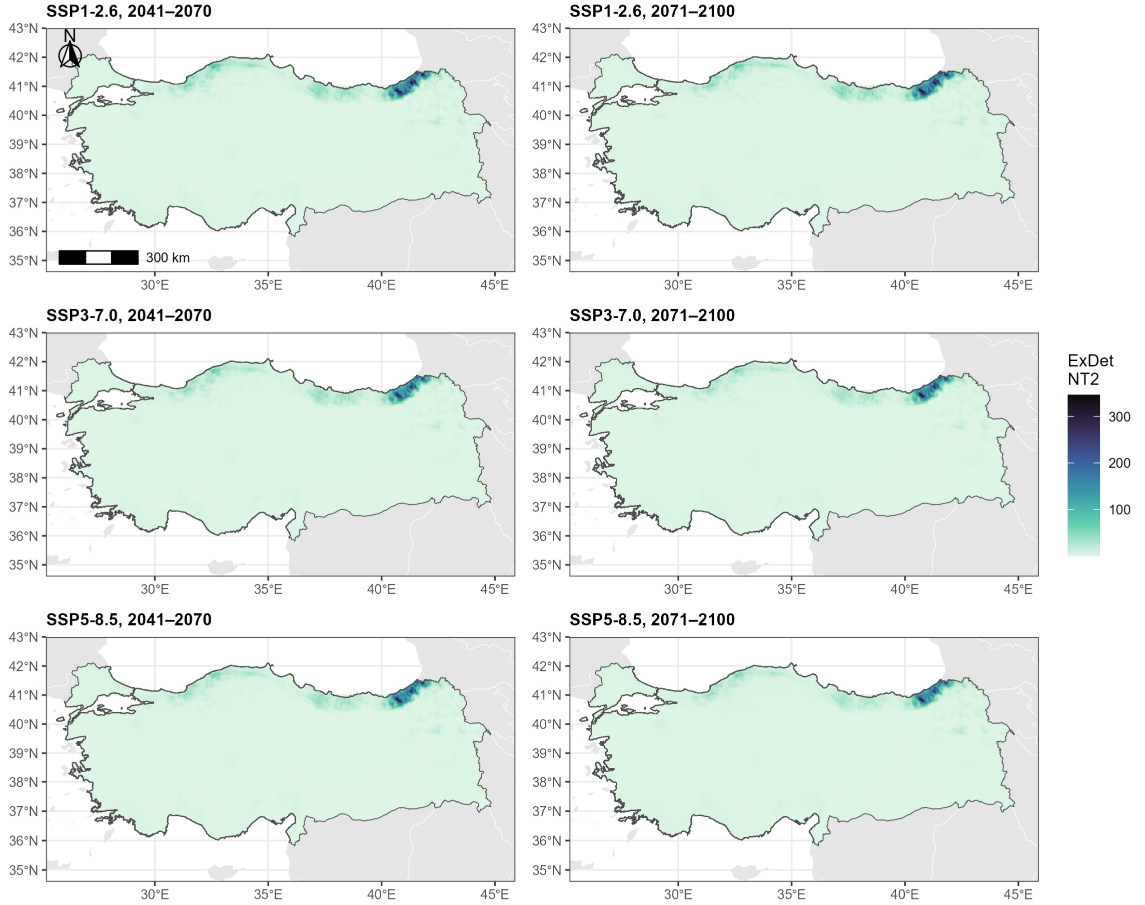

Figure S12. ExDet NT2 surface. Extrapolation–detection NT2 surface for each scenario–period combination, averaged across the five GCMs. NT2 quantifies novel combinations of predictors (combinatorial novelty), complementing the univariate MESS (Figure S11). Higher values mark climate where projections are least reliable.

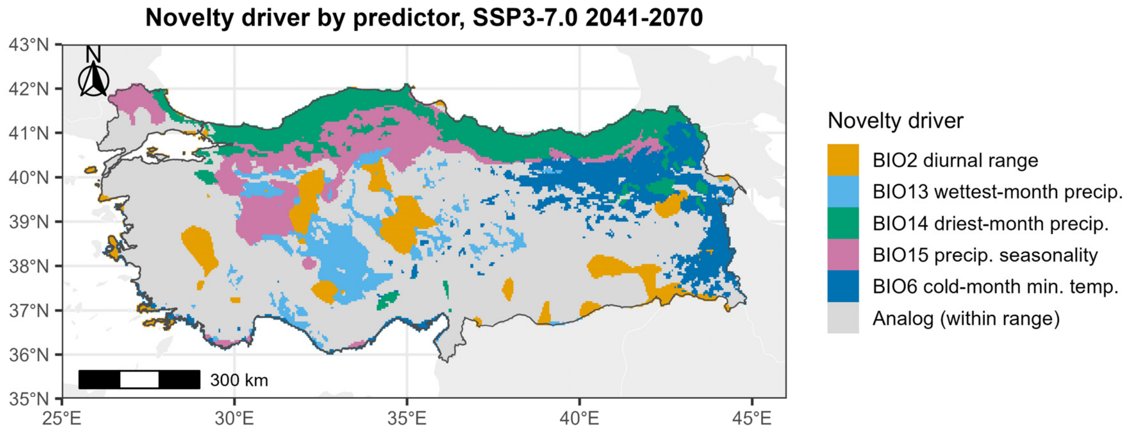

Figure S13. Predictor driving climatic novelty for the SSP3-7.0, 2041–2070 five-GCM mean projection of *Oena capensis* across Türkiye. Each non-analogue cell (MESS < 0) is coloured by its most dissimilar predictor. Cells within the calibration range are grey. Colours follow the colourblind-safe Okabe-Ito palette. The mapped non-analogue area reflects univariate (MESS) novelty on the ensemble-mean climate and is therefore a subset of the total non-analogue fraction reported in the Results, which additionally counts combinatorial (ExDet NT2) novelty and per-GCM extremes.

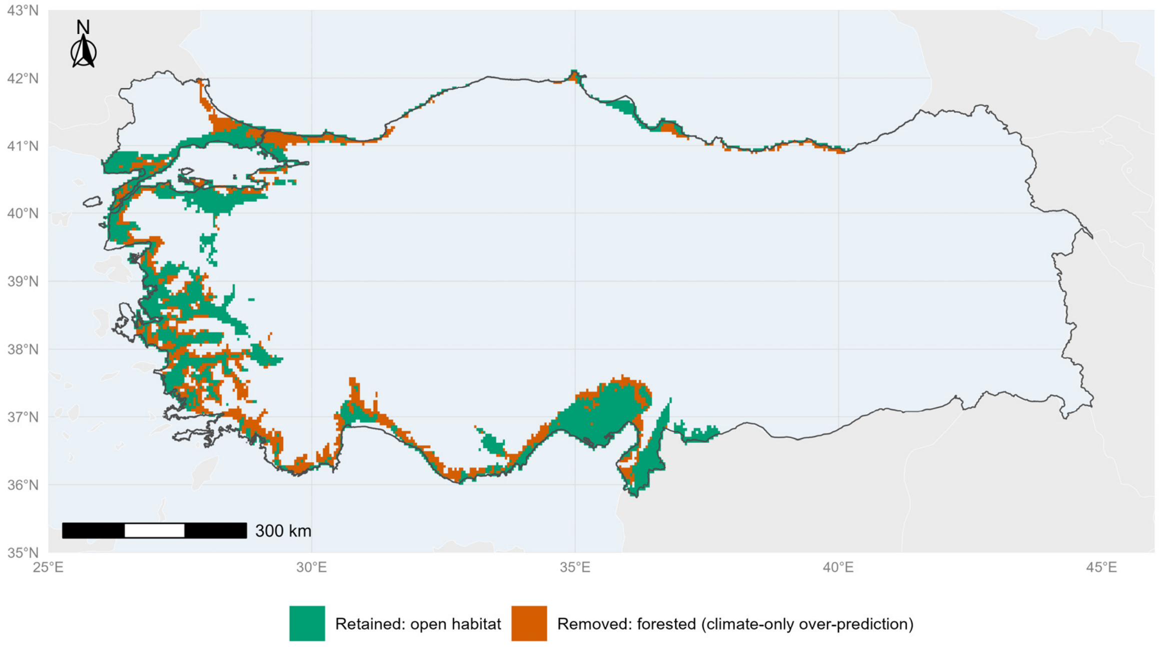

Figure S14. AOH refinement. Current climatic suitability of *Oena capensis* in Türkiye split into open habitat retained (bluish-green) and dense forest removed (vermilion, ESA WorldCover tree-cover ≥ 0.5); in the latter, the climate-only model over-predicts. Sensitivity to the tree-cover threshold is reported in Table S12.

Supplementary Tables

Table S1. ODMAP (overview, data, model, assessment, prediction) protocol for the *Oena capensis* ensemble model, following Zurell et al. [32].

Section	Field	Value
Overview	Authorship	B. Kabasakal
Overview	Model objective	Predict current and future climatic suitability; quantify niche dynamics between native and colonised ranges
Overview	Focal taxon	*Oena capensis* (Namaqua Dove)
Overview	Location	Calibration (accessible area M): Sub-Saharan Africa, SW Arabia, Mediterranean Basin; projection: Türkiye
Overview	Scale of analysis	2.5 arcmin (~5 km)
Overview	Hypotheses	Climatic suitability is constrained chiefly by precipitation and cold tolerance; the colonised range conserves the native climatic niche
Overview	Assumptions	Occurrences approximate the realised climatic niche; target-group background represents the accessible area; the retained predictors capture the main climatic constraints
Overview	Software	R 4.5; biomod2 4.3, ecospat 4.1, terra 1.9, sf 1.1, ENMeval, usdm, CoordinateCleaner, spThin
Data	Biodiversity data source	GBIF + expert literature records
Data	Response type	Presence-only (target-group background)
Data	Predictor source	CHELSA v2.1 bioclim (Karger et al. 2017)
Data	Spatial resolution	2.5 arcmin (~5 km)
Data	Temporal context	Climate normal 1981–2010 (current projections); occurrence records screened to the modern period before thinning
Data	Data cleaning	CoordinateCleaner (7 tests) + MAD outlier removal + 5 km spatial thinning
Model	Algorithms	GLM, GAM, GBM, MAXNET, ANN, CTA, MARS (7 retained; down-sampled random forest RFd failed to fit and was excluded)
Model	Variable selection	VIF < 10 (usdm::vifstep); BIO6 retained a priori as a cold-tolerance constraint
Model	Pseudo-absence strategy	Random, 10,000 pseudo-absences per run, 5 runs
Model	Ensemble method	Weighted-mean ensemble (EMwmean), em.by = PA + run
Model	Tuning	ENMeval block cross-validation for MAXNET (fc = L, rm = 5.0)
Model	Thresholding	MaxSSS = 0.490; 10th-percentile training presence (P10) = 0.155
Assessment	Cross-validation	5-fold random split (80/20), 5 pseudo-absence runs
Assessment	Performance metrics	True Skill Statistic (TSS), AUC, continuous Boyce index (ensemble CBI = 0.818)
Assessment	Spatial autocorrelation	Residual Moran's I over the calibration (M) extent: ~0.55 at 0-100 km (dnearneigh) and ~0.76 at k = 8 nearest neighbours, declining to ~0.27 at ~490 km (correlogram); spatially structured residuals acknowledged in the limitations
Assessment	Plausibility check	Predicted current suitability is consistent with known occupied areas; response curves are ecologically interpretable
Assessment	Extrapolation diagnostics	MESS (univariate, NT1) and ExDet NT2 (combinatorial) computed per scenario
Prediction	Future scenarios	SSP1-2.6, SSP3-7.0, SSP5-8.5
Prediction	GCMs and periods	MPI-ESM1-2-HR, MRI-ESM2-0, IPSL-CM6A-LR, GFDL-ESM4, UKESM1-0-LL; periods 2041–2070 and 2071–2100
Prediction	Uncertainty quantification	Across-model SD and CV (Figure S7), inter-GCM SD (Figure S10), and the per-scenario non-analogue fraction (Table S9)
Prediction	Niche dynamics	ecospat COUE: D = 0.104, expansion = 0.003, stability = 0.997, unfilling = 0.155

Table S2. Sample size and prevalence for the *Oena capensis* models.

Quantity	Value
Native presence (thinned)	18,432
Presence (thinned)	694
Total presence	19,126
Background pool	6,213
Pseudo-absence sets per run	5

Quantity	Value
Pseudo-absences per set	10,000
Nominal prevalence [presences / (presences + PA)]	0.2767

Table S3. Sensitivity of the niche-conservatism inference for *Oena capensis* to native-sample rarefaction and to a geometric null, and native-to-colonised niche-transfer metrics. Values are means with 2.5–97.5% ranges across 100 replicates where applicable.

Analysis	Metric	Value
Rarefaction (native to 689)	Niche stability	0.999 (0.999–1.000)
Rarefaction (native to 689)	Niche unfilling	0.177 (0.158–0.199)
Rarefaction (native to 23)	Niche stability	0.948 (0.771–1.000)
Geometric null (random colonised, 689)	Niche stability	0.992 (0.989–0.995)
Observed	Niche stability	0.997
Transfer (native to colonised)	Continuous Boyce index	-0.65 (-0.81, -0.38)
Transfer (native self-fit)	Continuous Boyce index	0.79
Transfer	Colonised inside native envelope	99.7%
Transfer	Colonised above native P10	89.0%
Transfer	Median native suitability (colonised/background)	0.057 / 0.106

Table S4. Per-algorithm sensitivity, specificity, True Skill Statistic (TSS), and area under the receiver operating characteristic curve (AUC), reported as cross-validation means. The down-sampled random forest (RFd) is not shown. All its sub-models failed during fitting because the down-sampling size exceeded the per-partition presence count, so it produced no per-algorithm metrics and was excluded from the ensemble.

Algorithm	Sensitivity	Specificity	TSS	AUC
CTA	82.4	84.5	0.645	0.881
ANN	81.5	81.8	0.629	0.886
GBM	81.7	81.7	0.623	0.892
MARS	78.9	80.6	0.593	0.867
GAM	75.8	83	0.586	0.868
GLM	76.3	81.6	0.58	0.813
MAXNET	71.8	84.4	0.561	0.854

Table S5. Predictors retained for *Oena capensis* and their ensemble permutation importance (mean and standard deviation). Predictors were selected with a variance-inflation-factor criterion (VIF < 10). BIO6 was retained a priori as a northern cold-tolerance constraint.

Predictor	Description	Mean importance	SD	Selection
BIO13	Precipitation of the wettest month	0.385	0.099	VIF < 10
BIO14	Precipitation of the driest month	0.331	0.151	VIF < 10
BIO6	Minimum temperature of the coldest month	0.279	0.078	VIF < 10 (a priori retained)
BIO2	Mean diurnal temperature range	0.227	0.097	VIF < 10
BIO15	Precipitation seasonality (coefficient of variation)	0.158	0.126	VIF < 10

Table S6. Threshold-choice sensitivity. The current suitable area for *Oena capensis* across Türkiye under the maximum sensitivity plus specificity threshold (MaxSSS = 0.490) and the 10th-percentile training-presence threshold (P10 = 0.155).

Threshold	Cutoff	Suitable area (km²)	% of Türkiye
MaxSSS (max sensitivity + specificity)	0.49	91,699	11.7
P10 (10th-percentile training presence)	0.155	289,158	36.9

Table S7. Niche-dynamics metrics for the comparison between the native and colonised ranges of *Oena capensis*, computed within the COUE framework in principal-component space. See Table S3 and Figure S3 for sensitivity and native-model transfer metrics.

Metric	Value	Interpretation
Schoener's D	0.104	Low niche overlap
Warren's I	0.284	Low niche overlap
Equivalency test (p)	0.000999	Niches not equivalent
Similarity native to colonised (p)	0.686	Not more similar than random
Similarity colonised to native (p)	0.0729	Not more similar than random
Expansion	0.003	Niche outside the native niche
Stability	0.997	Colonised niche within native-occupied conditions
Unfilling	0.155	Native niche not yet occupied in the range

Table S8. Gain, loss, and stable suitable area (km²) for *Oena capensis* across Türkiye under three SSP scenarios and two periods, averaged across five GCMs. The net change is the gain minus the loss. Loss and stable areas differ slightly from Table 2, which is computed per GCM (threshold-then-average). Table S8 uses the GCM ensemble-mean projection.

SSP	Period	Stable (km²)	Loss (km²)	Gain (km²)	Net change (km²)
SSP126	2041–2070	90,172	968	55,141	54,173
SSP126	2071–2100	89,858	1,282	83,956	82,674
SSP370	2041–2070	90,696	444	110,743	110,299
SSP370	2071–2100	90,128	1,012	196,812	195,801
SSP585	2041–2070	90,613	527	139,498	138,970
SSP585	2071–2100	90,035	1,105	250,318	249,213

Table S9. Non-analogue climate fraction for each scenario, period, and GCM, quantified as the percentage of the projection surface with negative multivariate environmental similarity (MESS < 0). The complementary combinatorial-novelty view (extrapolation-detection NT2) is shown in Figure S12.

SSP	Period	GCM	Non-analogue (%)
SSP126	2041–2070	MPI-ESM1-2-HR	74.2
SSP126	2041–2070	MRI-ESM2-0	75
SSP126	2041–2070	IPSL-CM6A-LR	71.3
SSP126	2041–2070	GFDL-ESM4	79.8
SSP126	2041–2070	UKESM1-0-LL	72.5
SSP126	2071–2100	MPI-ESM1-2-HR	75.1
SSP126	2071–2100	MRI-ESM2-0	77
SSP126	2071–2100	IPSL-CM6A-LR	71.3
SSP126	2071–2100	GFDL-ESM4	83.1
SSP126	2071–2100	UKESM1-0-LL	74.3
SSP370	2041–2070	MPI-ESM1-2-HR	71.8
SSP370	2041–2070	MRI-ESM2-0	75.2
SSP370	2041–2070	IPSL-CM6A-LR	71.9
SSP370	2041–2070	GFDL-ESM4	78.6
SSP370	2041–2070	UKESM1-0-LL	75.5
SSP370	2071–2100	MPI-ESM1-2-HR	73.2
SSP370	2071–2100	MRI-ESM2-0	85.7
SSP370	2071–2100	IPSL-CM6A-LR	70.7
SSP370	2071–2100	GFDL-ESM4	79.4
SSP370	2071–2100	UKESM1-0-LL	84.7
SSP585	2041–2070	MPI-ESM1-2-HR	73.1
SSP585	2041–2070	MRI-ESM2-0	78.3
SSP585	2041–2070	IPSL-CM6A-LR	71.5

SSP	Period	GCM	Non-analogue (%)
SSP585	2041–2070	GFDL-ESM4	78.7
SSP585	2041–2070	UKESM1-0-LL	81.3
SSP585	2071–2100	MPI-ESM1-2-HR	95.2
SSP585	2071–2100	MRI-ESM2-0	86.6
SSP585	2071–2100	IPSL-CM6A-LR	75.6
SSP585	2071–2100	GFDL-ESM4	82.8
SSP585	2071–2100	UKESM1-0-LL	85.4

Table S10. Leading-edge northward expansion of *Oena capensis* within Türkiye. The regression rate, the endpoint rate, and the number of years with records are reported descriptively. A fitted-trend confidence interval is not reported because of the small number of years (n = 12). The endpoint rate is negative while the regression rate is positive, reflecting the noise in a short, twelve-year endpoint comparison relative to the fitted trend.

Quantity	Value
Regression rate (km/yr)	2.44
Endpoint rate (km/yr)	-0.4
Years with Türkiye records (n)	12

Table S11. Optimal parameter–geographical-detector interaction matrix for the spatial pattern of predicted suitability within Türkiye. q is the joint power of each predictor pair. This table provides the values visualised in Figure 8b.

Predictor pair	q (first)	q (second)	q (joint)	Interaction
BIO2 × BIO6 (mean diurnal range × min temp coldest month)	0.227	0.576	0.795	Enhance, bivariate
BIO15 × BIO6 (precip seasonality × min temp coldest month)	0.178	0.576	0.701	Enhance, bivariate
BIO14 × BIO6 (precip driest month × min temp coldest month)	0.041	0.576	0.682	Enhance, nonlinear
BIO13 × BIO6 (precip wettest month × min temp coldest month)	0.031	0.576	0.675	Enhance, nonlinear
BIO2 × BIO15 (mean diurnal range × precip seasonality)	0.227	0.178	0.525	Enhance, nonlinear
BIO2 × BIO14 (mean diurnal range × precip driest month)	0.227	0.041	0.403	Enhance, nonlinear
BIO2 × BIO13 (mean diurnal range × precip wettest month)	0.227	0.031	0.281	Enhance, nonlinear
BIO13 × BIO15 (precip wettest month × precip seasonality)	0.031	0.178	0.253	Enhance, nonlinear
BIO14 × BIO15 (precip driest month × precip seasonality)	0.041	0.178	0.203	Enhance, bivariate
BIO13 × BIO14 (precip wettest month × precip driest month)	0.031	0.041	0.098	Enhance, nonlinear

Table S12. Area of habitat (AOH) sensitivity to forest-cover threshold for *Oena capensis* in Türkiye (ESA WorldCover tree-cover fraction).

Tree-cover threshold	Climate (km^2)	AOH (km^2)	Removed forest (km^2)	Removed (%)	Removed north (km^2)	Removed south (km^2)
0.3	91,699	45,195	46,504	50.7	6,413	21,162
0.5	91,699	61,855	29,844	32.5	4,960	13,939
0.7	91,699	77,978	13,720	15	3,021	6,564

Table S13. Sensitivity of the recorded versus unfilled monitoring classification to the record neighbourhood aggregation distance for *Oena capensis* in Türkiye. At each distance, climatically suitable cells (MaxSSS threshold) lying within that distance of a confirmed Türkiye occurrence (n = 23) are classified as recorded and those beyond it as unfilled. The total climatically suitable area is 91,699 km^2.

Neighbourhood distance (km)	Recorded (km^2)	Unfilled (km^2)	Unfilled (%)
10	1,744	89,955	98.1
20	5,229	86,469	94.3
30	9,108	82,591	90.1
50	16,811	74,887	81.7
